# Nonadiabatic Vibrational Resonance Raman Spectra from Quantum Dynamics Propagations with LVC Models. Application to Thymine

**DOI:** 10.1021/acs.jpca.2c05271

**Published:** 2022-09-13

**Authors:** Qiushuang Xu, Daniel Aranda, Martha Yaghoubi Jouybari, Yanli Liu, Meishan Wang, Javier Cerezo, Roberto Improta, Fabrizio Santoro

**Affiliations:** † School of Physics and Optoelectronics Engineering, Ludong University, 264025 Yantai, Shandong, PR China; ‡ School of Physics Engineering, Qufu Normal University, 2673100 Qufu, Shandong, PR China; ¶ Consiglio Nazionale delle Ricerche, Istituto di Chimica dei Composti Organo Metallici (ICCOM-CNR), SS di Pisa, Area della Ricerca, via G. Moruzzi 1, I-56124 Pisa, Italy; § Instituto de Ciencia Molecular (ICMol)., Universidad de Valencia, c/Catedrático José Beltrán, 2, 46980 Paterna, Spain; ∥ Departamento de Química, Universidad Autónoma de Madrid, 28049 Madrid, Spain; ⊥ Consiglio Nazionale delle Ricerche, Istituto di Biostrutture e Bioimmagini (IBB-CNR), Via De Amicis 95, I-80145 Napoli, Italy; # DTU Chemistry, Technical University of Denmark, Kemitorvet Bldg 207, DK-2800 Kongens Lyngby, Denmark

## Abstract

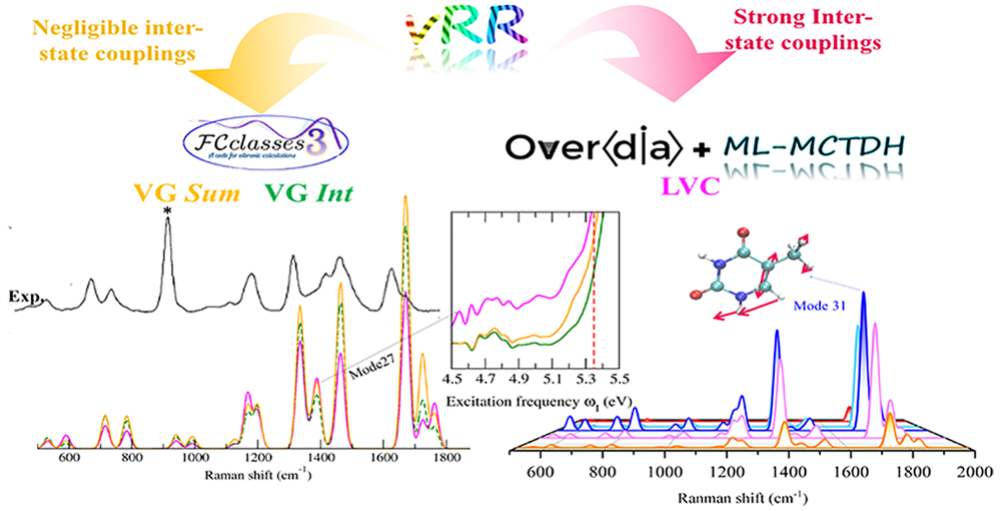

We present a viable protocol to compute vibrational resonance Raman (vRR) spectra for systems with several close-lying and potentially coupled electronic states. It is based on the parametrization of linear vibronic coupling (LVC) models from time-dependent density functional theory (TD-DFT) calculations and quantum dynamics propagations of vibronic wavepackets with the multilayer version of the multiconfiguration time-dependent Hartree (ML-MCTDH) method. Our approach is applied to thymine considering seven coupled electronic states, comprising the three lowest bright states, and all vibrational coordinates. Computed vRR at different excitation wavelengths are in good agreement with the available experimental data. Up to 250 nm the signal is dominated by the lowest HOMO → LUMO transition, whereas at 233 nm, in the valley between the two lowest energy absorption bands, the contributions of all the three bright states, and their interferences and couplings, are important. Inclusion of solvent (water) effects improves the agreement with experiment, reproducing the coalescence of vibrational bands due to CC and C=O stretchings. With our approach we disentangle and assess the effect of interferences between the contribution of different quasi-resonant states to the transition polarizability and the effect of interstate couplings. Our findings strongly suggest that in cases of close-lying and potentially coupled states a simple inclusion of interference effects is not sufficient, and a fully nonadiabatic computation should instead be performed. We also document that for systems with strong couplings and quasi-degenerate states, the use of HT perturbative approach, not designed for these cases, may lead to large artifacts.

## Introduction

Vibrational resonance Raman (vRR)^1−3^ is a powerful spectroscopic technique to study the properties of molecular excited states. When the excitation energy is close to resonance with the transition energy between the ground state (GS) and an excited state (ES), the contribution of such state to the vibrational transition polarizability is strongly enhanced and possibly dominates all the other ones. In these situations, for a bright resonant ES, the intensity of the vRR band of a specific mode is ruled by the Franck–Condon (FC) integrals between GS and ES vibrational states. This means that such intensity depends primarily on the displacement of the initial and final state equilibrium geometries along such a mode, but it can also be affected by quadratic-terms differences like frequency changes and Duschinsky mixings.

Different effective time-independent (TI)^4−8^ and time-dependent (TD)^9−12^ methods have been proposed to compute vRR in these situations, considering (i) a single resonant ES (ii) with negligible couplings with other ESs. In the following, we will refer to these approaches as “single-state” ones.

In many molecules, the ES manifold is rather dense so that more than one ES can be in quasi-resonance with the excitation wavelength. In these cases, they all contribute to the transition polarizability and interferences can take place. Assuming that the ES states are not coupled, this situation can still be addressed by computing separately the transition polarizability for each resonant ES, and then summing them up before computing the vRR intensities.^13^

On the other side, when the electronic states are quasi-degenerate, even small interstate couplings can alter their photophysical and spectroscopic behavior. It is thus interesting to investigate, and indeed, it is one of the goals of this study, how the interplay between interferences and couplings can affect the vRR signal.

To do that, in this contribution we will resort to a fully nonadiabatic approach based on quantum-dynamical (QD) wave packet propagations on coupled potential energy surfaces (PES). We will use the multiconfiguration TD Hartreee (MCTDH)^14,15^ method and, in particular, its multilayer extension (ML-MCTDH).^16−18^ Such propagation techniques are extremely efficient for model Hamiltonians where diabatic states have harmonic PES and the couplings are described by low-order Taylor expansions in the normal coordinates. In particular, we will focus on the linear vibronic coupling (LVC) model, which assumes that normal models and frequencies of all coupled states coincide with the ground-state ones and considers that interstate couplings are linear functions of the normal coordinates.^19,20^ In fact, we have recently worked out an effective and general approach based on a maximum-overlap diabatization to parametrize LVC models on the grounds of time-dependent density functional theory (TD-DFT) calculations.^21,22^ Here we will show that, by combining such fast parametrization of LVC models with ML-MCTDH propagations, it is nowadays possible to compute the vRR of systems with several coupled excited states and dozens of normal modes.

Here, as a test case we consider a DNA nucleobase: thymine, Thy. All nucleobases are heteroatom ring structures substituted with functional groups, either a carbonyl or an amino group conjugated with the π system. As a result, they all exhibit close-lying bright and dark states with different properties.^23,24^ The latter, together with the existence of easily accessible conical intersections with the GS are responsible for the rich photophysics exhibited by nucleobases,^23−27^ and their ability to effectively dissipate the energy absorbed by UV radiation,^23,24^ which is potentially harmful and may lead to photodamage.^23,28^ Because of these features, nucleobases are expected to be a good playground to test the computational approach we propose, and to investigate the interplay between interference and interstate couplings in determining the vRR spectra. From a complementary point of view, this study will further assess the potentialities of vRR spectroscopy in disentangling the complex photophysical paths operative in nucleobases.^29−35^

We will thus compute the vRR spectra of Thy, considering all normal coordinates (39) and a large number of possibly interacting states (7). To test the accuracy of the developed strategy, we will also consider the limit case in which interstate couplings are set to zero. In this case, the LVC Hamiltonian collapses on the so-called Vertical Gradient (VG) model.^36^ For “single-state” approaches and harmonic PESs, the correlation functions necessary for a TD computation of vRR have an analytical expression,^37−40^ and we rederived and implemented them in our freely available code FCclasses3.^41^ We will also use FCclasses3 to investigate the effects of Duschinsky mixings and frequency changes with the Vertical and Adiabatic Hessian (VH and AH) models,^36,42^ comparing their predictions with LVC and VG models in which such effects are neglected. These analytical correlation functions can be derived at both the Franck–Condon (FC) and the Herzberg–Teller (HT) level. The HT perturbative theory^43^ actually allows us to introduce the effects of weak interstate couplings through the linear dependence of the transition dipoles on the nuclear coordinates, and it is currently included in the most-advanced TI^44^ and TD single-state methods for vRR spectroscopy.^38,40^ By using the new implementation in FCclasses3, we here investigate the differences between the perturbative HT and nonperturbative LVC approaches and show that, as for absorption,^45,46^ the application of the HT approach for close-lying states with remarkable couplings, i.e., beyond the limits it was conceived for, can give rise to large errors for vRR spectroscopy.

Finally, we investigate solvent effects on the vRR spectra by performing some key calculations both in the gas phase and in water solution, described with implicit solvent models.^47^

## Theory

Let us consider a system with a ground state |*g*⟩ and a set of coupled electronic diabatic states |*d*_*i*_⟩. We use a LVC Hamiltonian in the dimensionless normal coordinates **q** (and associated momenta **p**) of the ground state,
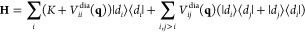
1The kinetic (*K*) and potential (*V*) terms are
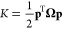
2
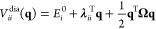
3
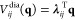
4where **Ω** is the diagonal matrix of the vibrational frequencies of state *g*.  is the *i*th excited-state energy at the *g* equilibrium geometry, **λ**_*ii*_ is the energy gradient of state *i* and accounts for a shift of the equilibrium position, and **λ**_*ij*_ is the gradient of the interstate coupling . Therefore, , the PES of diabatic state *i*, is a quadratic function of **q** that shares the same normal modes and frequencies of *g* and the interstate couplings  are linear functions of **q**.

Let us consider the following (spectral) representation for the electric dipole moment
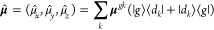
5

Because we are using a basis set of diabatic states, ideally independent of the coordinates, the vectors **μ**^*gk*^ can be considered constant (FC approximation). The key quantity to compute the vRR spectra from the ground vibrational state ν_0_ to the final vibrational state ν_*f*_, both associated with the electronic state *g*, is the transition polarizability tensor
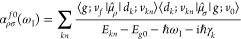
6where ρ and σ indicate the Cartesian components, ω_I_ is the excitation frequency, *E*_*g*0_ is the energy of the initial state, and the sum is over all the possible (quasi-)resonant vibronic states |*d*_*k*_; *v*_*kn*_⟩ with energy *E*_*kn*_ and lifetime γ_*k*_. From now on, such a lifetime will be considered independent of *k* (γ). We now use the following equivalence
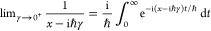
7to obtain
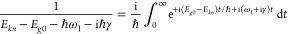
8and therefore, a TD expression of the polarizability tensor
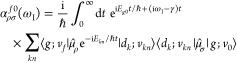
9

Exploiting the fact that *∑*_*kn*_|*d*_*k*_; *v*_*kn*_⟩⟨*d*_*k*_; *v*_*kn*_| = 1 we get
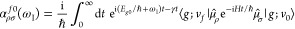
10

Once the transition polarizability is obtained, the vRR intensity can be computed as follows:^2,44^
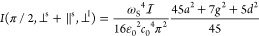
11where the rotational invariants *a*, *g*^2^, and *d*^2^ are
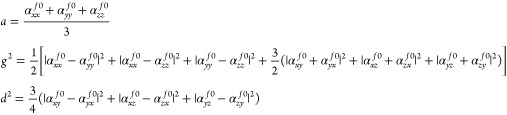


In the next section, we describe more in detail the practical protocol we follow to compute the vRR spectra.

### Computational Protocol

Let us introduce a more explicit notation for the vibrational states |*v*_*f*_⟩ = |**0** + 1_*f*_⟩, where we clarify that it is a state with 1 quantum on mode *f* and 0 quanta on all other modes. The ground vibrational state is |*v*_0_⟩ = |**0**⟩. The vRR spectrum can be computed according to the following steps

(1) For each bright state |*d*_*m*_⟩, we start a propagation of a wavepacket obtained vertically exciting the ground vibrational state of the ground state: i.e., |Ψ(0)⟩ = |*d*_*m*_; **0**⟩.
(2) For each bright diabatic state |*d*_*k*_⟩ and each vibrational final state |*v*_*f*_⟩ = |**0** + 1_*f*_⟩, we compute the cross-correlation with the bra state ⟨*d*_*k*_; **0** + 1_*f*_|,
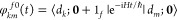
13
(3) We compose the 3 × 3 tensor of the total correlation function
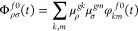
14which corresponds to the bracket in eq 10.
(4) We perform the Fourier transform for each (ρ, σ) component
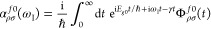
15where we introduced the Lorentzian damping with width γ.
(5) We compute the intensity according to eq 11.

It is noteworthy that, in the limiting case in which the resonant diabatic states are uncoupled, for each |Ψ(0)⟩ = |*d*_*m*_; **0**⟩ the only nonvanishing contributions to the total correlation in eq 14 are for *k* = *m*. Still,  and, therefore, the polarizability tensor α^*f*0^ is the sum of the contributions of the different resonant states *m*. This means that interferential (*Int*) effects can arise when squares are taken to compute the rotational invariants. The effect of these interferences was analyzed in detail for pyrene by some of us in ref (36). To assess the relevance of these interferential effects, here we defined an additional protocol in which the computation of the vRR intensity is simply repeated by considering just one state per time, and then the resulting values are summed (*Sum*).

It is worthy to notice that for vanishing interstate couplings the LVC model collapses into the VG one.^36^

To summarize this section, when considering more than one quasi-resonant electronic state, we shall compute the vRR intensities by using three different protocols:

- **LVC**. It includes the effects of both interstate couplings and the interference of the different states.
- **VG*Int****.* It neglects the effects of interstate couplings but considers the interference of the different states, i.e., a sum is taken in eq 14 over all the relevant states *k* (*k* = *m*).
- **VG*Sum****.* It neglects the effects of both interstate couplings and interferences. The vRR intensity is thus simply the sum of what is obtained by repeating the computation *k* times, each of them considering only the ES state *k* in eq 14. Afterward, the vRR intensities in eq 11 are summed for all relevant states.

As described in detail elsewhere,^45,48^ the absorption (ABS) spectrum can also be formulated as the Fourier transform of (auto)correlation functions, obtained by placing the ground vibrational state at both sides of the bracket in eq 13. The effect of the interstate couplings on the absorption spectra has been here investigated with LVC calculations, which again reduces to VG*Sum* when couplings are neglected.

### Computation Details

**Figure 1:**
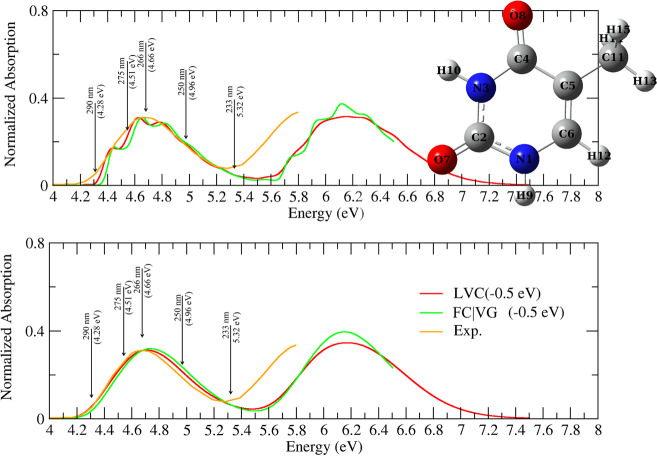
Absorption spectra of thymine computed by FC|VG or LVC models, convoluted with a Gaussian of HWHM = 0.04 eV (top) or 0.12 eV (bottom). Experimental data, in water, from ref (29). Arrows indicate the excitation wavelength used in the vRR experiments in ref (29).

Electronic calculations were performed with Gaussian16 package of programs,^49^ adopting density functional theory (DFT) level for GS properties and TD-DFT for excited states. The CAM-B3LYP functional in combination with the 6-31G(d) and 6-311G+(d,p) basis set was adopted. The chemical structure of thymine, optimized with *C*_*s*_ symmetry, is shown in Figure 1.

The effect of water solvent was simulated by the PCM (polarizable continuum model) with the linear response implementation,^47,50^ in the nonequilibrium regime for the calculation of the vertical transitions and in the equilibrium one for vibronic ABS and vRR calculations.

After the vRR spectra were computed, for a better comparison with experiments GS vibrational frequencies were scaled by a typical factor 0.96 so to correct for the inaccuracy of the adopted electronic structure theory and for the lack of anharmonic corrections. In the following, if not otherwise specified, we shall report scaled frequencies. QD propagations with ML-MCTDH method were performed with the Quantics code,^51,52^ using a variable mean field (VMF) with a RungeKutta integrator of order 5 and accuracy 10^–7^ for both ABS and vRR spectra. We considered the lowest lying bright states and the close-lying dark states. This choice practically includes the first three ππ*, two nπ*, and two *πRy*_σ_, and we adopted TD-DFT to parametrize the corresponding LVC models. For further analysis, spectra were also computed by applying the TD calculation implemented in FCclasses3^41^ at 0K based on analytical time-correlation functions. The relevant formulas are reported in the Supporting Information. For these calculations, we adopted VG model, which like LVC neglects the Duschinsky effect and changes of the normal frequencies with the electronic state. To investigate their impact, we also employed VH (the PES of final state is expanded around the initial equilibrium geometry) and AH (the PES of final state is expanded around its own equilibrium geometry) harmonic models.^36^

As far as the transition dipole is considered, because electronic states in LVC are diabatic, i.e., because they are ideally independent of the nuclear coordinates, the FC approximation can be invoked in these calculations. For “single state” approaches, though, the considered electronic states are adiabatic and in these cases we considered both the FC and FC+HT approximations.

For ABS, we apply a Gaussian broadening with half-width at half-maximum (HWHM) of 0.04 and 0.12 eV. We compute vRR intensities as 2D functions depending on the incident frequency ω_I_ and the Raman shift ω_I_ – ω_S_. The damping constant, γ (see eq 6), was set to either 0.04 or 0.12 eV. The smaller value 0.04 eV was selected because it is very close to the estimated value of the homogeneous contribution to the line width, given in ref (29) (0.044 eV). On the contrary, as shown below, the larger value, 0.12 eV, gives a reasonable reproduction of the experimental absorption shape, thus accounting phenomenologically also for the inhomogeneous broadening. We notice that, formally speaking, homogeneous and inhomogeneous contributions should be added in a two step procedure as done in ref (29). However, results would be similar for what concerns the focus of our paper, and therefore, we avoided this more involved approach. In the conclusions, we comment on possible future developments to include solvent broadening effects in a nonphenomenological way. For better visualization, all the vRR stick spectra have been further convoluted along the Raman shift coordinate with a Gaussian line shape, with HWHM equal to 15 cm^–1^ (when not stated differently).

For a fair comparison between computed and experimental vRR bands, it is necessary to approximately reproduce in the computations the resonance conditions investigated in the experiment. To do that, we first measure the shift of the maxima of the calculated and experimental ABS spectra  and then for an experimental vRR at the excitation frequency , we obtain the excitation to be adopted in the computations  with the following formula: .

Further tests reported in the Supporting Information show that, when we switch off the interstate couplings, the spectra obtained with a numerical propagation with ML-MCTDH are virtually indistinguishable from those computed with analytical correlation functions by FCclasses3.^41^ For this reason, in the following, we shall label simply as “VG” the spectra obtained with LVC setting to zero the couplings.

## Results

Table 1 reports the lowest energy excited states in the gas phase at the FC point, according to CAM-B3LYP/6-311G+(d,p) calculations. The first excited state is a dark nπ* state followed by a bright HOMO → LUMO bright state, ππ_1_^*^. At significantly higher energies, we find a second nπ* state (S_4_) and two close-lying bright states, ππ_2_^*^ (S_5_) and ππ_3_^*^ (S_6_). S_3_ and S_7_ have a Rydberg character. The situation is similar to the smaller basis set 6-31G(d) (see Table S1 in the Supporting Information), except for a ∼0.2 eV destabilization of the ππ* states and for a much larger one of Rydberg states. Confirming previous findings,^23,26,27,53^ as reported in Table S2, inclusion of bulk solvent effects (water) with PCM^54^ leads to a small red shift of ππ_1_^*^ and to a larger blue shift of n_O_π_1_^*^, which, as a consequence, invert their relative stability in the FC point.

**Table 1:** Symmetry, Vertical Excitation Energies *E*_*gf*_ (eV), Oscillator Strengths (δ_OPA_) of the First Seven Excited States for Thymine, Calculated with CAM-B3LYP and the 6-311G+(d,p) Basis Sets in the Gas Phase

	6-311G+(d,p)					
state	sym	*E*_*gf*_ (eV)	δ_OPA_	trans	char	coeff
S_1_	A′	5.14	0.00	H–1 → L	n_O_π_1_^*^	0.63
S_2_	A′	5.31	0.19	H → L	ππ_1_^*^	0.69
S_3_	A″	5.94	0.0006	H → L+1	π*Ry*_σ_1__	0.69
S_4_	A″	6.47	0.00	H–1 → L+4	n_O_π_2_^*^	0.40
				H–3 → L+4		0.38
S_5_	A′	6.67	0.055	H–2 → L	ππ_2_^*^	0.69
S_6_	A′	6.73	0.22	H → L+4	ππ_3_^*^	0.66
S_7_	A″	6.78	0.0013	H → L+3	π*Ry*_σ_2__	0.60

### Absorption Spectra

The vibronic absorption spectra (adopting both LVC and VG models) computed in the gas phase are compared with the experimental spectrum in water^29^ in Figure 1. The computed spectra are blue-shifted by ∼0.5 with respect to the experimental ones (first band), and the absorption intensity in the valley at 5.2–5.4 eV is underestimated, mainly because of an overestimation (∼0.3 eV) of the energy gap between the two lowest energy absorption bands (associated with ππ_2_^*^ and ππ_3_^*^). These discrepancies are partially because of solvent effects. As discussed in detail in the Supporting Information, inclusion of bulk solvent effects red-shifts the computed spectra by 0.1–0.2 eV and decreases the energy gap between the two lowest energy bands by ∼0.1 eV.

The lack of Duschinsky rotation and frequency changes is another source of error. These effects can be accounted for with AH or VH calculations (neglecting interstate couplings) leading to a red shift of the ππ_1_^*^ band of ∼0.1 eV (Figure S8 in the Supporting Information). Computations with VH and AH are not feasible for higher lying states because, as a result of interstate couplings, they feature too many modes with imaginary frequencies. The remaining difference between experiments and calculations is because of inaccuracies of the electronic calculations. In any case, considering the scope of the present study and that a meaningful comparison can only be performed by reproducing resonance conditions similar to those in the experiment (shown as arrows in Figure 1), the vRR spectra have been computed at excitation frequencies blue-shifted by 0.5 eV. Moreover, when discussing the spectra for an excitation at 233 nm (in the valley), we performed test calculations by applying an additional red shift to ππ_2_^*^ and ππ_3_^*^ (see below).

Nonadiabatic effects can be analyzed by comparing the results of the LVC and FC|VG models, and they are only moderate at high resolution (HWHM = 0.04 eV) and almost completely washed out at low resolution (HWHM = 0.12 eV). Interestingly, such large broadening leads to a band shape that agrees with the experimental one.

### Vibrational Resonance Raman Spectra

**Figure 2:**
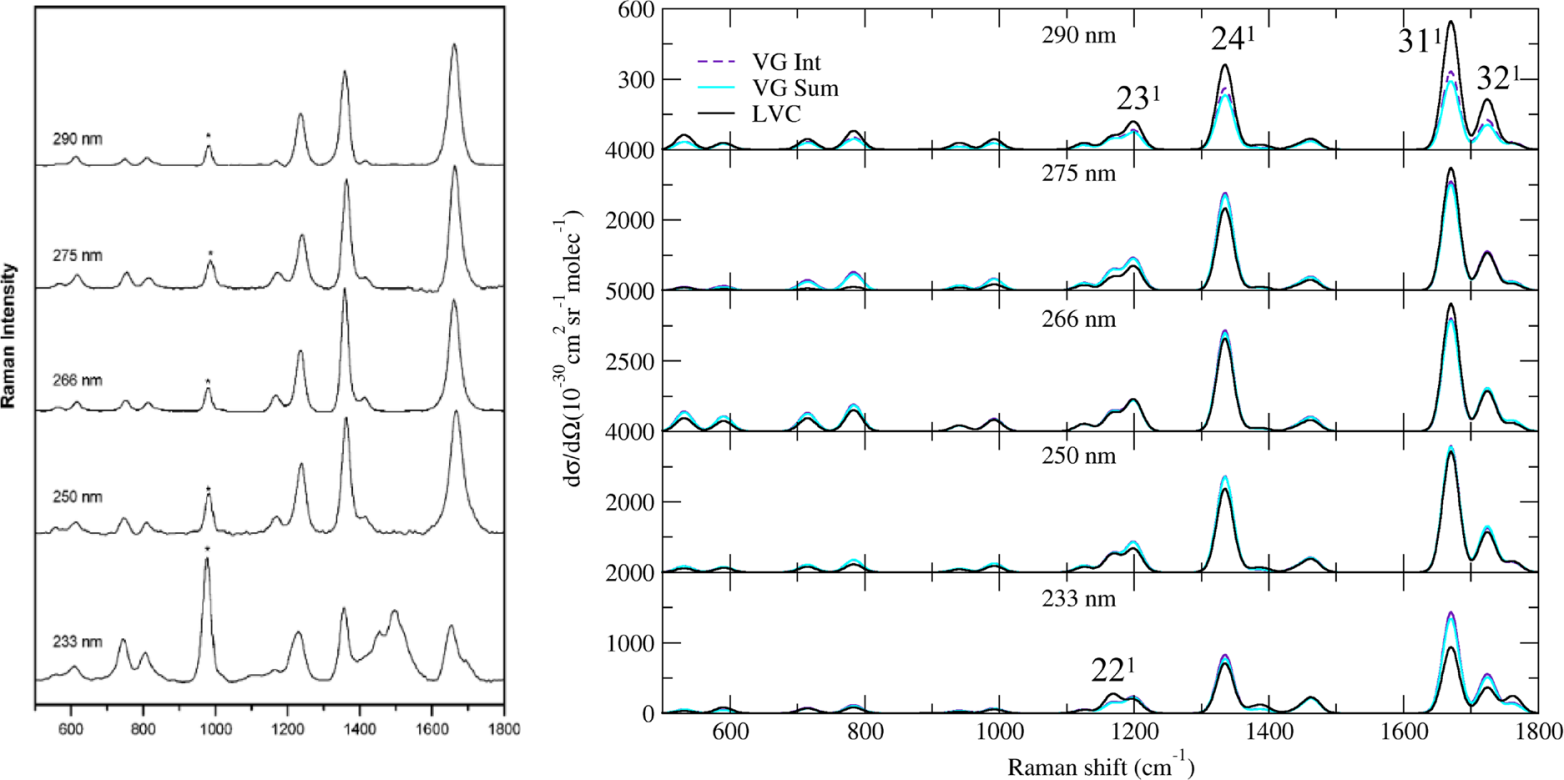
Right panel: Computed vibrational resonance Raman spectra of thymine convoluted with a Lorentzian with damping γ = 0.04 eV. In the left panel, we report the experimental data in aqueous solutions. Reprinted with permission from ref (29). Copyright 2007 American Chemical Society. The experimental band marked with an asterisk is attributed to the internal standard.

**Figure 3:**
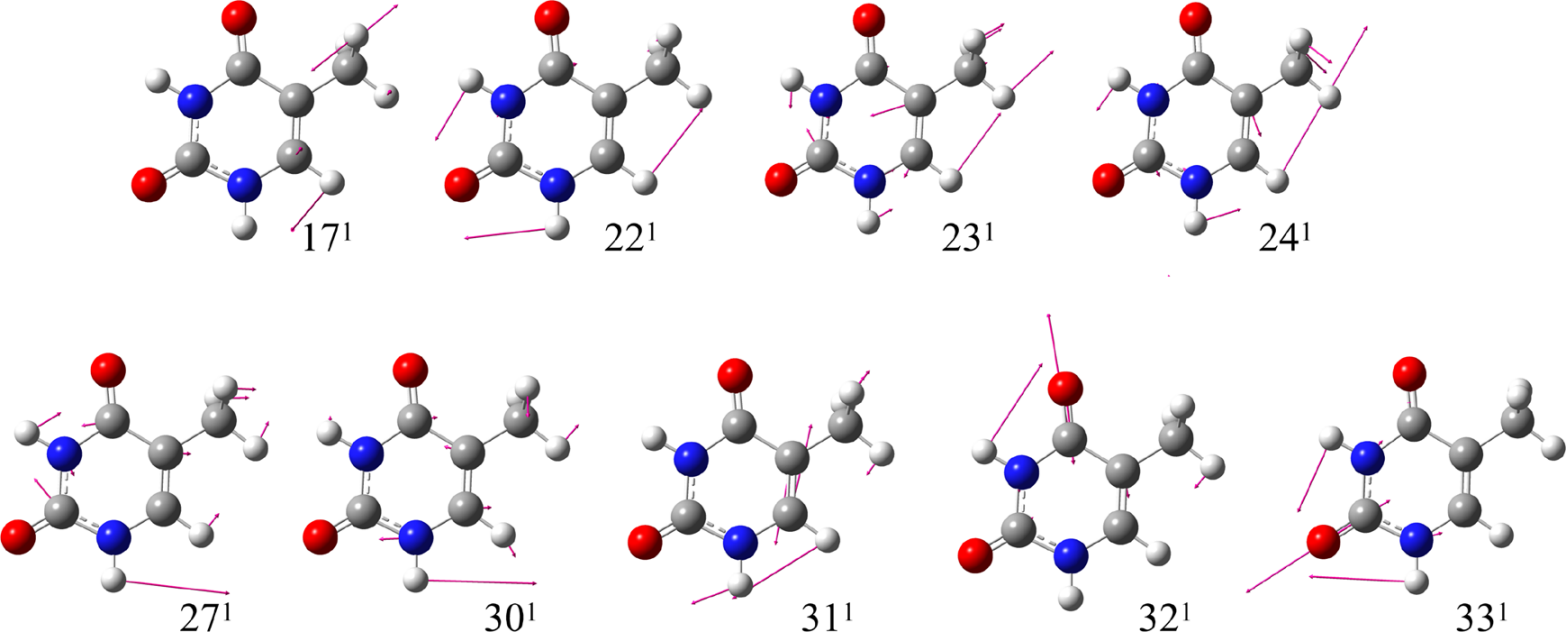
Schematic representation of the most relevant vRR-active vibrational modes of thymine.

In our approach we compute the nonadiabatic LVC vRR spectrum as a 2D signal, in terms of the excitation frequency ω_I_ and the Raman shift (see an example in Figure S1 in the Supporting Information). Typical 1D vRR spectra are then obtained as a section of the 2D signal at a given value of ω_I_. Raman excitation profiles, on the contrary, are 1D cuts for a specific Raman shift, and therefore, they are functions of ω_I_. As mentioned above, calculations were performed with a damping constant, γ, of 0.04 and 0.12 eV. Note that this results in a Lorentzian broadening with HWHM = γ along ω_I_. In both cases, similar vRR spectra are obtained, whereas the Raman excitation profiles are of course smoother (see Figure S6 in the Supporting Information). Although we have shown that a larger broadening provides absorption spectra in better agreement with experiment, in the following, we mainly discuss vRR spectra with γ = 0.04 eV, because a higher resolution allows us to grasp more easily the differences between the computational models. In Figure 2, we report the computed and the experimental vRR spectra at excitation wavelengths λ = 290, 273, 266, 250, and 233 nm. Our calculations nicely reproduce the experimental line shapes, which are quite similar up to 250 nm. The most intense bands are at 1200, 1335, 1671, and 1725 cm^–1^. They can be assigned to the fundamentals of modes 23^1^, 24^1^, 31^1^, and 32^1^ sketched in Figure 3 and corresponding to NH and CH bending, a CC stretching, and a CO stretching.

For excitation frequencies up to 250 nm, it is sufficient to consider the resonance with only the three lowest energy excited states. Figure S7 shows indeed that the LVC results obtained with 7 or 3 states are equivalent. Moreover, nonadiabatic and interferential effects are quite small, as indicated by the close similarity of the results obtained with the three protocols LVC, FC|VG*Int*, and FC|VG*Sum* (Figure 2). The vRR profile is thus dominated by ππ_1_^*^, enabling us to focus on this state, with “single state” approaches, for a more in-depth analysis of our results.

One of the major discrepancies with respect to the experiment is observed above 1700 cm^–1^, where in the experimental spectrum only one strong band appears, to be compared with the three features present in the computed one (though the one at 1671 cm^–1^ is, by far, the most intense one). They correspond to the C5C6 (see atom labels on Figure 1) stretching (31^1^) and to the C4O8 stretching coupled with the bending C2N3H10 (32^1^), whereas the very weak shoulder (33^1^) is the C2O7 stretching mixed with C2N3H10 and C2N3H9 bendings. Duschinsky mixings and frequency changes are not responsible for the discrepancies between experimental and computational vRR spectra in this region. In fact, Figures S9–S11 of the Supporting Information show that the vRR spectra provided by VG, VH and AH models are similar, although VH and AH Raman excitation profiles are generally smoother than VG ones (Figure S11).

**Figure 4:**
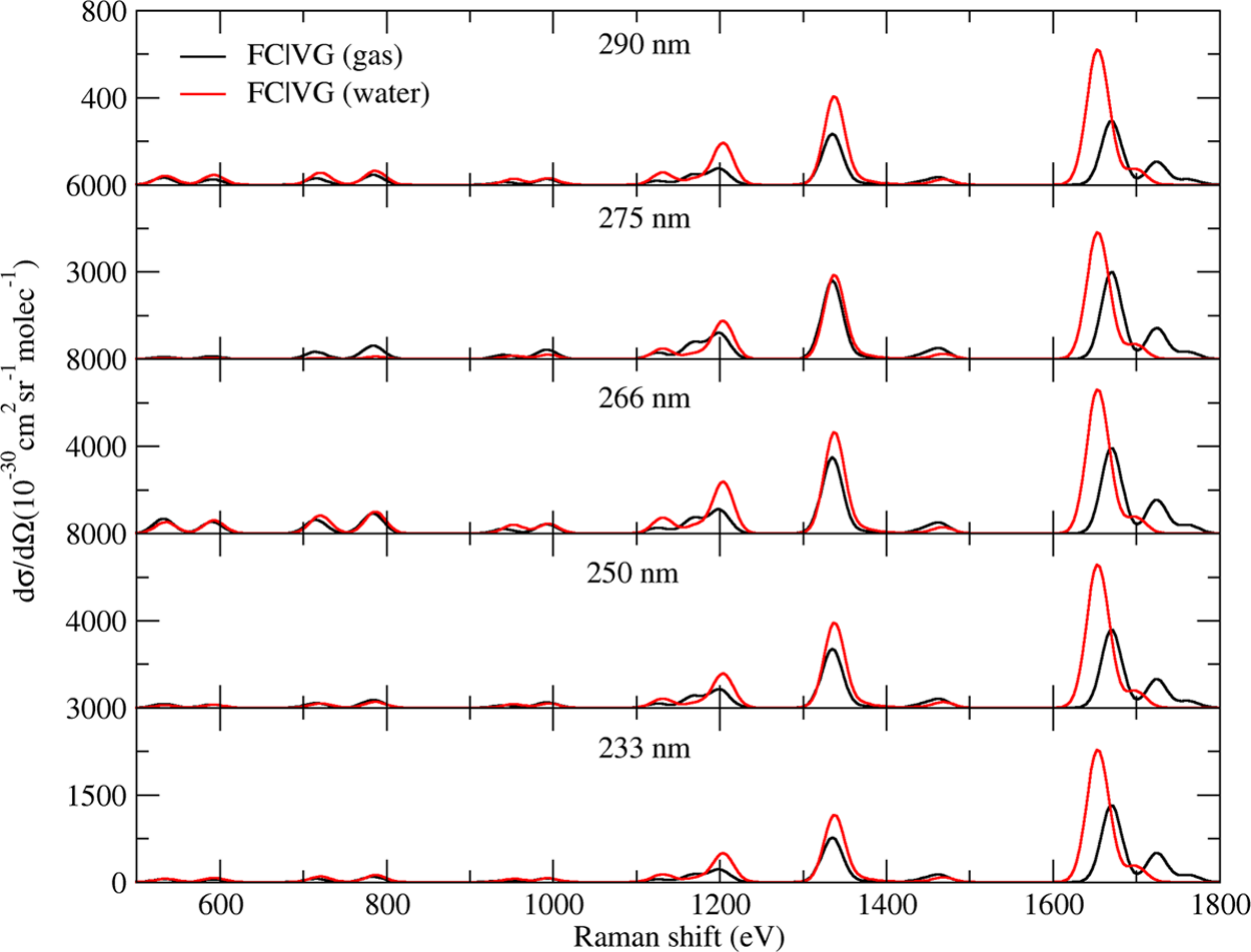
Vibrational resonance Raman spectra of thymine computed by single-state FC|VG model in vacuo and in water, considering only the contribution of ππ_1_^*^, with a damping γ = 0.04 eV.

Inclusion of solvent effects is instead crucial to improve the agreement, in that region, with experimental spectra measured in water. As shown in Figure 4, the single-state vRR spectra of ππ_1_^*^ computed in water and in the gas phase are very similar, except for the bands >1600 cm^–1^, where for computations in water only one feature is present in much closer agreement with the experiments. As detailed in Figure S23 of the Supporting Information, in water mode 32 and mode 31 become very close in frequencies (and the relative intensity of the former increases), coalescing in a single band.

In Section S5.3 of the Supporting Information, we investigate the dependence of the results in water on the scaling factor α of the sphere radii adopted to build up the PCM cavity (default α = 1.1). The effect is negligible for the shape of the absorption spectrum and small for their position. Concerning the vRR spectra, the effect is small for most of the bands. A partial exception is the band >1600 cm^–1^ discussed above, because the CO stretching is particularly sensitive to the effects. Therefore, for the small value α = 1.0, its frequency red-shifts so much to separate from the CC stretching, causing a new splitting of the vRR band which worsens the agreement with experiment. Further details and plots of absorption spectra, vRR spectra at different excitation wavelengths, and Raman excitation profiles for different α values can be found in the Supporting Information.

### Closer Look at the vRR Spectrum at 233 nm

We now focus on the vRR spectrum recorded at 233 nm. As shown in Figure 2, at this wavelength a new experimental band at ∼1500 cm^–1^ appears, which is absent in the computed spectra, both in the gas phase and in water (see Figure 4). Actually, as shown in the Supporting Information, the FC|VG vRR spectra of the higher lying ππ_2_^*^ and ππ_3_^*^ states exhibit two new bands at ∼1400 and 1450 cm^–1^, assigned to the fundamentals of modes 27 and 30, which are sketched in Figure S12. However, their intensity is very small, because of the overestimation (by ∼0.3 eV) of the energy gap between these two states and the lowest energy band.

**Figure 5:**
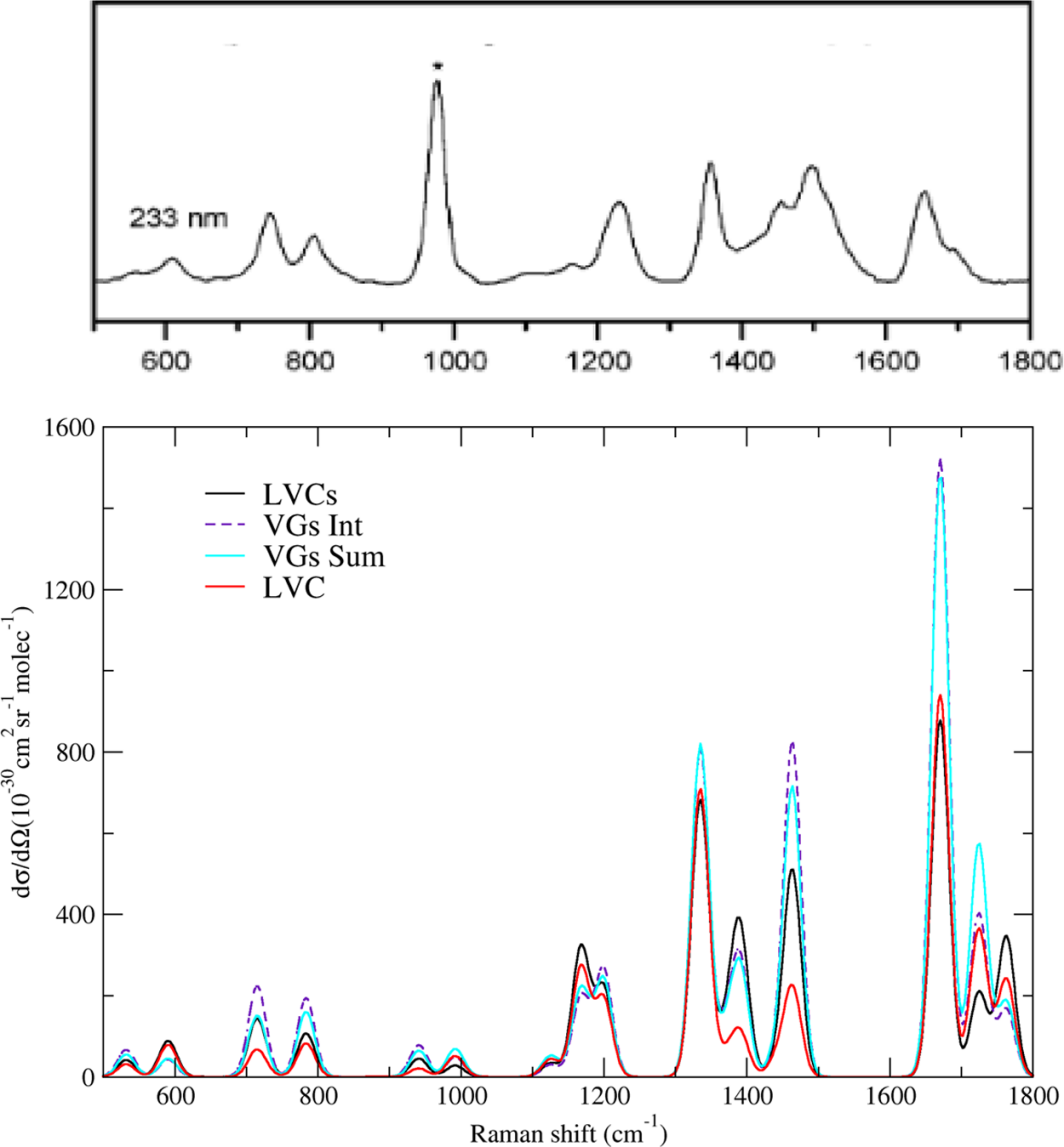
Bottom panel: vibrational resonance Raman spectra computed for thymine, including the lowest energy excited states and red-shifting by 0.3 eV ππ_2_^*^ and ππ_3_^*^ with respect to the other states, at the LVC (LVCs) and at FC|VG level (VCs*Int* and VGs*Sum*), for an excitation frequency corresponding to 233 nm in the experiment. The LVC vRR spectrum computed without red-shifting ππ_2_^*^ and ππ_3_^*^ is shown for comparison. Top panel: experimental vRR spectrum of thymine in water, following an excitation at 233 nm. Reprinted with permission from ref (29). Copyright 2007 American Chemical Society.

In Figure 5, we show the LVC vRR spectra computed after artificially red-shifting ππ_2_^*^ and ππ_3_^*^ by ∼0.3 eV with respect to the three lowest energy excited states (LVCs). Two new intense bands appear at ∼1450 cm^–1^ (stronger) and ∼1400 cm^–1^, in good agreement with the experimental peak at 1500 cm^–1^ and the shoulder at 1450 cm^–1^. Shifting ππ_2_^*^ and ππ_3_^*^, even the FC|VGs*Int* model predicts the existence of these two bands (where the “s” label recalls that the shift has been applied). However, the relative intensity of the two bands computed by LVCs is much closer to the experimental one. This finding indicates that the coupling between the three lowest energy bright states modulate the spectroscopic vRR signal. The differences between *Int* and *Sum* profiles in Figure 5 clearly highlight that interference has a visible but modest effect, whereas comparison of both results with LVCs ones shows that the impact of interstate couplings is much larger. Further analysis on the relative impact of interstate couplings and interferences is reported in the next section.

### Deeper Analysis of Interferential and Nonadiabatic Effects

**Figure 6:**
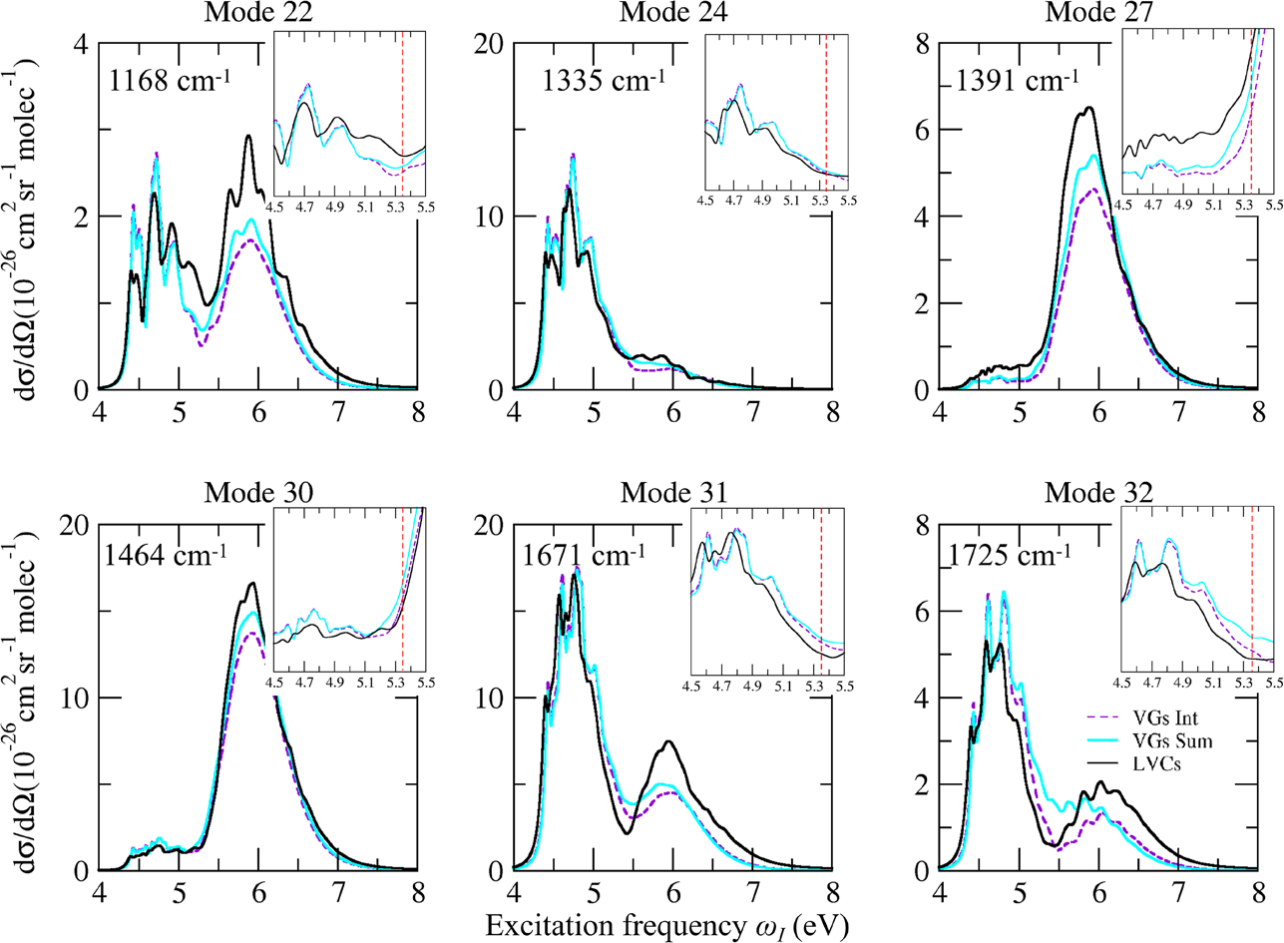
Raman excitation profile of six modes of thymine computed including the seven lowest energy excited states and red-shifting ππ_2_^*^ and ππ_3_^*^ by 0.3 eV with respect to the other states, at the LVC (LVCs, black), FC|VGs*Int* (purple), and FC|VGs*Sum* (cyan) levels, with a damping γ = 0.04 eV. The excitation frequencies adopted in the computations to reproduce the experimental resonance conditions are here red-shifted by 0.5 eV. In this way, they match the experimental ones. In particular, the dashed vertical lines in the insets indicate the excitation at 5.32 eV (233 nm).

To analyze more in detail the effect of interstate interferences and couplings, we selected six modes corresponding to the most intense bands 22^1^, 24^1^, 27^1^, 30^1^, 31^1^, and 32^1^, and in Figure 6 we compare their Raman excitation profile computed at the nonadiabatic LVC level with those computed by the FC|VG*Int* and FC|VG*Sum* models. We consider here the computations in which ππ_2_^*^ and ππ_3_^*^ have been red-shifted by 0.3 eV (the same analysis without the shift is reported in Figure S15 in the Supporting Information). Comparison of FC|VGs*Sum* and FC|VGs*Int* indicates that interferential effects are present, but they are always modest, except for band 32^1^ and ω_I_ ∼5.3 eV. This region corresponds to excitations between the two absorption bands, where the interference is large and destructive (*Int* intensities are much smaller than *Sum* ones). As shown by the comparison between LVCs and FC|VGs*Int* results, the most interesting finding is that nonadiabatic couplings play a much larger role than the interference. In addition to a small loss of the vibronic resolution, nonadiabatic effects change the intensity, mostly in the region at ∼6.0 eV and at 5.2–5.5 eV. In the former region, corresponding to the second absorption band, the intensity predicted by LVC is always larger. Interestingly, when ππ_2_^*^ and ππ_3_^*^ are not shifted, LVC gives the opposite prediction (see Figure S15 in the Supporting Information), indicating that interstate couplings affect the vRR intensity in a quite complex way. In the valley between the two bands, at 5.2–5.5 eV, LVC is more intense for band 22^1^ but less intense for both modes 31^1^ and 32^1^. Interestingly, only in some cases does FC|VG*Int* seem to partially capture the difference between LVC and FC|VG*Sum*, indicating that such a difference is because of interferential effects (check, for example, the Raman profile of band 32^1^ at ∼5.5 eV). In several other cases, FC|VG*Int* predictions are not even intermediate between FC|VG *Sum* and LVC (check, for instance, the band 27^1^ at ∼6.0 eV). Because LVC is the “correct” result, we have to conclude that in these cases FC|VG*Int* is changing FC|VG *Sum* in the wrong direction. These findings highlight that, for close-lying and potentially coupled states, one should not rely on the simple *Int* regime and perform calculations like LVC where the effect of couplings is fully taken into account. This conclusion is confirmed by the spectra obtained including only the three bright states in the LVC model (see Figure S14), which are also different with respect to VG*Int* predictions.

### Contribution of Dark Electronic States

**Figure 7:**
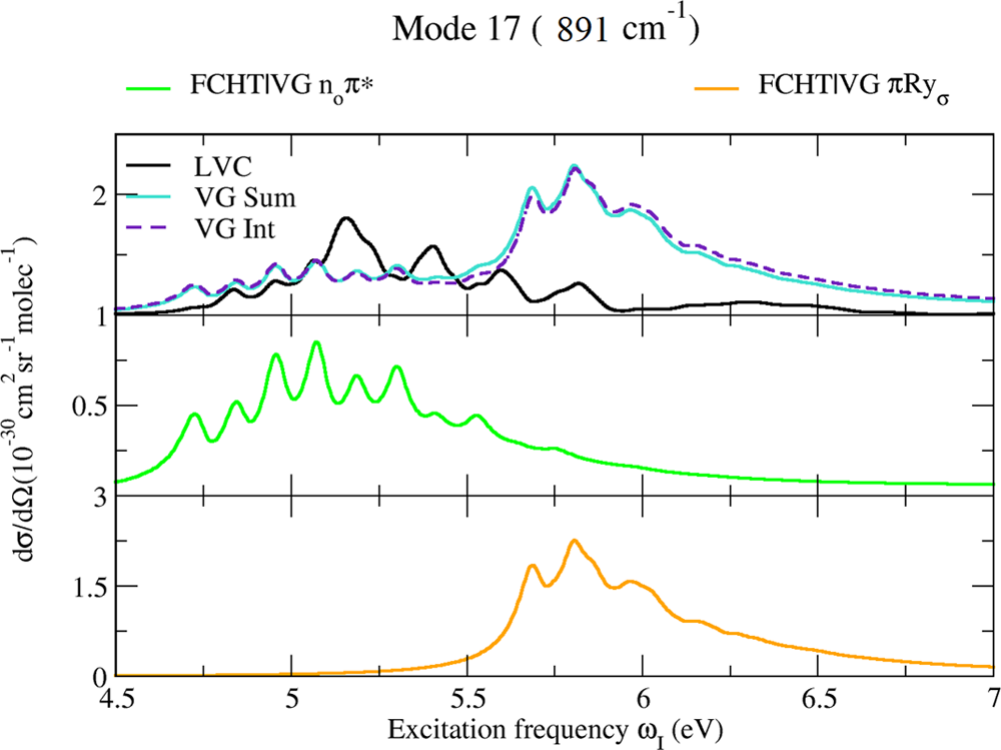
Raman excitation profile of A″ mode 17 of thymine, computed by the LVC model or different possible combinations of FC|VG and FCHT|VG models, considering the three lowest energy excited states and applying a damping γ = 0.04 eV.

Interstate couplings are expected to strongly enhance the contribution of the dark states. In a perturbative HT treatment, this is easily shown by comparing FC|VG and FCHT|VG predictions for the n_0_π* and *πRy*_σ_. Figure S19 in the Supporting Information documents that, at the FCHT level, new bands appear and other bands increase their intensity by ∼10^3^ or even 10^6^. However, at least for A′ modes, these bands remain so much weaker than those resulting from the bright states that, in practice, the contribution of dark states cannot be observed. The situation is quite different for the weak signals associated with A*″* modes. In fact, their fundamentals are Raman-active only thanks to the nonadiabatic couplings, and according to symmetry, they arise only from the contribution of weak A*″* states. It is interesting to notice that, considering different possible protocols within the perturbative approach, this prediction is respected only if we adopt the FCHT approximation only for the weak states and rely on a FC approach for the bright ones. Vice versa, Figure S19 in the Supporting Information documents a dramatic failure of the FCHT approach if it is used also for the bright state ππ_1_^*^. In fact, this protocol predicts a nonphysical and dramatic increase of the Raman signal, which is not observed if we properly apply a nonperturbative method like LVC. Such artifacts have already been described in the calculation of absorption spectra.^45,46^ In Figure 7, we consider the fundamental of one of the A*″* modes with the largest HT effect, mode 17 (sketched in Figure 3), and we compare its Raman excitation profile as predicted by LVC, VG*Int*, and VG*Sum*. We focus on excitations in the first absorption band and include only the contributions of the three lowest energy excited states. VG*Int* and VG*Sum* results are practically identical, and therefore, interferential effects are negligible. Their intensity correctly reproduces the order of magnitude of the LVC predictions. However, the spectral shapes provided by LVC and VG models are quite different. The HT approach overemphasizes the contribution of n_O_π* below ∼4.75 eV and of *πRy*_σ_ above ∼5.5 eV, whereas it underestimates the contribution of n_O_π* in the range 4.75 eV < ω_I_ < 5.5 eV. Moreover, looking at the vibronic structure, it is possible to trace a one-to-one correspondence for most of the VG and LVC peaks but not for all of them (check, for example, at ∼5.2 eV). This finding shows that, when states are very close in energy, HT perturbative predictions should be treated with caution. In any case, the predicted intensity of these A*″* modes is much weaker than that of the A′ ones, and therefore, their experimental detection is very unlikely.

## Concluding Remarks

In this contribution, we presented a viable route to compute vRR spectra for semirigid systems with several close-lying and potentially coupled electronic states. It is based on LVC models and effective ML-MCTDH propagations. The LVC model is parametrized with respect to TD-DFT calculations with a quite general diabatization technique based on a maximum-overlap criterium that we implemented recently^21^ and interfaced with Gaussian 16.^55^

Our QD approach is similar to that presented by Stock and co-workers years ago^56^ and applied to pyrazine, within a reduced model including two electronic states and seven normal modes. We here show that, by exploiting the recent availability of the very effective ML-MCTDH propagations and the fast LVC parametrization with our approach,^21,55^ it is possible to treat a system like thymine considering all vibrations and a large number (here 7) of coupled states.

Moreover, to get additional insights on the different effects modulating the vRR signal, here we complement the LVC analysis with computations based on analytical expressions of the time-correlation functions, for the limiting cases in which interstate couplings become vanishingly weak. We also compare the predictions of the nonperturbative LVC calculations with those of perturbative HT approaches. Concerning the effects of the coexistence of several quasi-degenerate states for thymine, we investigate to what extent they operate through simple interference or to the fact that the states are actually coupled. Finally, we also get some insights on the role of environmental effects, by comparing the vRR signals computed in the gas phase and in solution.

We have applied our method to thymine, a prototypical heteroatomic ring, whose photophysical behavior is the focus of ongoing experimental and computational studies.^23,24,57−60^ The vRR spectra computed in the gas phase are in good agreement with the experimental ones, measured in water, for what concerns both the position and the relative intensity of the different peaks. The only significant discrepancy between the two sets of data concerns the high energy region (>1600 cm^–1^), where three peaks are present in the computed spectra, to be compared with the single experimental peak. This discrepancy can be corrected by including the solvent effect on the vibrational frequencies, which decreases the stretching frequencies of the CO bonds, leading to their coalescence with that of the CC double bond.

Our study shows that nonadiabatic effects are small for excitation frequencies in resonance with the first electronic band, which is dominated by the lowest bright transition ππ_1_^*^. For this band, frequency changes and Duschinsky mixings are also predicted to play a minor role, thus supporting the reliability of approaches (equivalent to VG) already adopted in the literature in ref (29).

To satisfactorily reproduce the experimental spectrum at 233 nm, corresponding to the valley between the first and second ABS band (due to the contribution of ππ_2_^*^ and, mostly, ππ_3_^*^), it was instead necessary to take into account the contributions of the three lowest bright states (ππ_1_^*^, ππ_2_^*^, and ππ_3_^*^). For vRR in this frequency region, we clearly document remarkable nonadiabatic effects, which are much larger than those from simple intereferences. Actually, we show cases in which accounting only for the interference can even change the theoretical predictions in the wrong direction. On this ground, for cases with close-lying states, we can strongly recommend performing a fully nonadiabatic computation.

Nonadiabatic effects play a much larger role on A″ modes, making them Raman active. However, their intensity is predicted to be too small to be detected. It also noteworthy that we show that improper usage of HT perturbative approach may lead to large artifacts.

Our study provides useful information on the potentialities of vRR for thymine and, more generally, for nucleobases, already a very active research field.^29−35^ Just to make an example, very recently Borrego-Varillas et al.,^58^ documented the involvement of a ring breathing mode at ∼750 cm^–1^ in the photoexcited dynamics, by adopting a pump–probe set up with sub-30 fs resolution. It is noteworthy that this same mode is actually also seen in the experimental vRR spectra (check Figure 2) and is reproduced by our calculations (it is mode 17 with a frequency of 891 cm^–1^). Both experimental and computed vRR spectra suggest, moreover, that its involvement should increase for higher excitation energies (233 nm) where also the higher ππ* states are involved. Our results also suggest that the coupling between ππ_1_^*^ and n_O_π* has a larger impact on the vRR of coupling modes with A″ symmetry. In particular, we analyzed the vRR signal of a mode corresponding to the out-of-plane mode of the H bonded to C6 atom, which couples the two states.^61^ However, we predict that its intensity is likely too small to make them visible in steady-state vRR experiments. It is very interesting to notice that, with their sub-30 fs experiments, Borrego-Varillas et al.^58^ observed the involvement of such a mode for uridine (where its frequency is smaller) and not in thymidine in water. In this context, we recall that the possible involvement of dark nπ* states in the photophysics of pyrimidines remains a lively debated issue, although for thymine in water it should be very small or even negligible.^53,58,62,63^

The measurement of vRR spectra for different excitation wavelengths is another useful tool to disentangle the role played by higher energy transitions. We have shown here that our calculations nicely reproduce how the vRR spectrum changes when ππ_2_^*^ and ππ_3_^*^ come into play. We have also shown that at higher excitation frequencies the intensity of the vRR spectrum is remarkably affected by the couplings between the bright states. In this context, it is worthy to notice that the inclusion of the contribution of the higher electronic states allows us to better reproduce the experimental asymmetry of experimental Raman excitation profiles reported for some bands in ref (29) with respect to the simulations performed in the same article considering only ππ_1_^*^ (check Figure S16). These findings suggest that it should be interesting to combine the information gained by vRR with those coming from time-resolved experiments.

Several developments of the approach presented here can be envisaged in the future. In particular, it would be important to tackle an inclusion of solvent effects with explicit models. In fact, because of its high polarity and its capability to establish hydrogen bonds, the effects of water solvent cannot be properly addressed with implicit models.^64^ Explicit water effects might impact the vRR spectra in several ways: not only changing the displacements along the normal modes and their ground-state frequencies or the relative stability of the excited states but also introducing broadening effects. The last two effects have been included only phenomenologically here and in the past.^29^ In the future, we will investigate the possibility to run the computation of vRR spectra in explicit solvent models, generalizing to resonance Raman the mixed quantum classical approach for absorption spectra in the condensed phase, which we proposed recently both for single-state^65^ and for nonadiabatic systems.^66^ It should be mentioned that, for vRR in explicit water models of systems with negligible nonadiabatic couplings, remarkable progress has been done recently by Gómez et al.,^67−69^ also accounting for the mutual solute/solvent polarization.

## Supporting Information Available

Derivation of the “single-state” analytical correlation functions for vRR for harmonic systems with negligible interstate couplings (FC and HT terms), absorption spectrum in the gas phase with the small 6-31G(d) basis set, further checks and additional plots of the computed vRR spectra with LVC and “single-state” approaches including also frequency changes and Duschinsky effects, excited states in water with PCM and additional computations of vRR spectra in water, also varying the α size cavity parameter (PDF)
