# Supplementary material for: Nonadiabatic Vibrational Resonance Raman Spectra from Quantum Dynamics Propagations with LVC Models. Application to Thymine
Source: J Phys Chem A. 2022 Sep 13;126(41):7468–79. doi: 10.1021/acs.jpca.2c05271 (PMC9596142; doi:10.1021/acs.jpca.2c05271)
Supplement: Supplementary file 1 — jp2c05271_si_001.pdf [file jp2c05271_si_001.pdf]

Supporting Information: Nonadiabatic Vibrational Resonance Raman from Quantum Dynamics Propagations with LVC models. Application to Thymine

Qiushuang Xu,[†,‡,¶] Daniel Aranda,[§] Martha Yaghoubi Jouybari,[¶] Yanli Liu,[†] Meishan Wang,[†] Javier Cerezo,[‖,¶] Roberto Improta,[⊥] and Fabrizio Santoro[*,¶]

† *School of Physics and Optoelectronics Engineering, Ludong University, 264025 Yantai, Shandong, PR China*

‡ *School of Physics Engineering, Qufu Normal University, 2673100, Qufu, Shandong, PR China*

¶*Consiglio Nazionale delle Ricerche, Istituto di Chimica dei Composti Organo Metallici (ICCOM-CNR), SS di Pisa, Area della Ricerca, via G. Moruzzi 1, I-56124 Pisa, Italy*

§ *ICMol, Universidad de Valencia, c/Catedrático José Beltrán, 2, 46980 Paterna, Spain*

‖ *Departamento de Química. Universidad Autónoma de Madrid, 28049 Madrid, Spain*

⊥*Consiglio Nazionale delle Ricerche, Istituto di Biostrutture e Bioimmagini (IBB-CNR), via Mezzocannone 16, I-80136 Napoli, Italy*

E-mail: fabrizio.santoro@pi.iccom.cnr.it

Contents

S1 Theory **S-4**

S1.1 Analytical correlation functions for vibrational resonant Raman spectroscopy for "single-state" harmonic systems . . . S-4

S1.1.1 Rayleigh band . . . S-9

S1.1.2 Fundamental bands . . . S-10

S2 An example of the full 2D nonadiabatic vibrational RR spectra computed with our protocol **S-12**

S3 Technical checks **S-13**

S3.1 FCclasses and ML-MCTDH deliver equivalent results for "single-state" cases . . S-13

S4 Additional results in gas phase **S-15**

S4.1 Effect of the basis set on the absorption spectrum. . . . S-15

S4.2 Effect of the broadening along ω_I on the vRR spectra . . . S-18

S4.3 Comparing LVC results with 3 and 7 states . . . S-18

S4.4 Single-state approaches for $\pi\pi_1^*$ . . . S-20

S4.4.1 Effect of different PES models. Predictions of VH and AH models for $\pi\pi_1^*$ S-20

S4.5 "Single-state" analysis of the effect of the $\pi\pi_2^*$ and $\pi\pi_3^*$ on the vRR spectrum . S-24

S4.6 LVC Raman excitation profiles for the six modes studied in the main text, without shifting $\pi\pi_2^*$ and $\pi\pi_3^*$ . . . S-26

S4.7 LVC Raman excitation profiles for three modes observed in the experiment . . . S-28

S4.8 Further tests on Herzberg-Teller effects . . . S-30

S4.8.1 The contribution of the two lowest excited dark states . . . S-30

S4.8.2 Comparison of LVC and FCHT|VG predictions for the A" mode 17 . . . . S-31

S5 Computations in water **S-33**

S5.1 Absorption spectrum with "single-state" approaches . . . S-34

S5.1.1 Contribution of $\pi\pi^*$ 1 in water S-34

S5.1.2 Adding the contribution of $\pi\pi^*$ 2 and $\pi\pi^*$3 in water S-35

S5.2 Vibrational resonance Raman spectra with "single-state" approaches S-37

S5.2.1 The contribution of $\pi\pi^*$ 1 in water S-37

S5.2.2 Effect of different PES models: Predictions of VH and AH models for $\pi\pi^*_1$ in water S-38

S5.2.3 Vibrational resonance Raman spectra in water adding the contribution of $\pi\pi^*$2 and $\pi\pi^*$3 . . . . S-40

S5.3 Tests of the impact of the PCM cavity-size parameter α on the absorption and resonance Raman spectra S-42

S5.3.1 Absorption spectrum of $\pi\pi^*$ 1 in water with different α S-42

S5.3.2 Vibrational resonance Raman spectra of $\pi\pi^*$ 1 in water with different α . S-44

S5.3.3 Raman excitation profiles of $\pi\pi^*$ 1 in water with different α S-46

References **S-49**

S1 Theory

S1.1 Analytical correlation functions for vibrational resonant Raman spectroscopy for "single-state" harmonic systems

For systems in which the initial i and final f states are not coupled to other states, and characterized by potential energy surfaces (PES) which can be described within harmonic approximation, the correlation functions necessary to compute the vibrational Resonance Raman (vRR) can be derived analytically. This is true also for models more general than VG, i.e. also accounting for frequency changes and Duschinsky mixings, described by the relation

$$\mathbf{Q}_i = \mathbf{J}\mathbf{Q}_f + \mathbf{K} \tag{S1}$$

We proposed to name the models accounting for such effects in a vertical or an adiabatic framework, respectively as Vertical Hessian (VH) and Adiabatic Hessian (AH).[S1]

In the following we report our derivation for the vRR correlation functions which has been implemented in the version 3.0 of the code FCclasses.[S2] The elements of the polarizability tensor at 0 K are

$$\alpha_{\rho\sigma}^{f0} = \frac{i}{\hbar^2}\int_0^{\infty} dt e^{+it\omega_I} e^{-it\Delta E/\hbar} e^{-\gamma_k t} \chi_{\rho\sigma}^{v_f^g}(t,T) \tag{S2}$$

where the following correlation function for the (ρ,σ) element was introduced,

$$\chi_{\rho\sigma}^{|\mathbf{v}_f^g\rangle}(t,T) = \langle \mathbf{v}_f^g|\mu_\rho^{gk} e^{-i\hat{H}_m t/\hbar} \mu_\sigma^{kg} e^{+i\hat{H}_g t/\hbar}|\mathbf{0}^g\rangle = e^{iE_0 t/\hbar}\langle \mathbf{v}_f^g|\mu_\rho^{gk} e^{-i\hat{H}_m t/\hbar} \mu_\sigma^{kg}|\mathbf{0}^g\rangle \tag{S3}$$

where $E_0 = \frac{1}{2}\hbar\sum_{i=1}^{N_{vib}}\omega_i$

If we now move to the coordinate representation and add two complete sets of normal mode

coordinates in the initial and final states,

$$\chi_{\rho\sigma}^{|\mathbf{v}_f^g\rangle}(t,T) = e^{iE_0t/\hbar}\int_{-\infty}^{\infty}\int_{-\infty}^{\infty}\int_{-\infty}^{\infty}\int_{-\infty}^{\infty} d\mathbf{Q}_f d\bar{\mathbf{Q}}_f d\mathbf{Q}_i d\bar{\mathbf{Q}}_i$$
$$\langle \mathbf{v}_f^g|\mathbf{Q}_i\rangle\langle\mathbf{Q}_i|\mu_\rho^{gk}|\mathbf{Q}_f\rangle\langle\mathbf{Q}_f|e^{-i\hat{H}_m t/\hbar}|\bar{\mathbf{Q}}_f\rangle\langle\bar{\mathbf{Q}}_f|\mu_\sigma^{kg}|\bar{\mathbf{Q}}_i\rangle\langle\bar{\mathbf{Q}}_i|\mathbf{0}^g\rangle \tag{S4}$$

The elements μ_ρ^{gk} and μ_σ^{kg} generally depend on nuclear coordinates, and they can be expanded as Taylor series in terms of $\mathbf{Q}_f$

$$\mu_\rho^{gk}(\mathbf{Q}) = \mu_\rho^{gk}(\mathbf{Q}_0) + \sum_{k=1}^{N_{vib}}\frac{\partial\mu_\rho^{gk}(\mathbf{Q})}{\partial Q_f}Q_f \equiv \mu_\rho^{gk,(0)} + \sum_{k=1}^{N_{vib}}\mu_{\rho,k}^{gk,(1)}Q_f \tag{S5}$$

where both the constant (FC) and linear (HT) terms are included, and a simplified notation to represent the first derivatives is introduced. Using the Duschinsky relation in Eq. S1 we can write:

$$\langle\mathbf{Q}_i|\mu_\rho^{gk}(\mathbf{Q}_f)|\mathbf{Q}_f\rangle = \mu_\rho^{gk}(\mathbf{Q}_f)\delta(\mathbf{Q}_i - \mathbf{J}\mathbf{Q}_f + \mathbf{K}) \tag{S6a}$$
$$\langle\bar{\mathbf{Q}}_f|\mu_\sigma^{kg}(\mathbf{Q}_f)|\bar{\mathbf{Q}}_i\rangle = \mu_\sigma^{kg}(\bar{\mathbf{Q}}_f)\delta(\bar{\mathbf{Q}}_i - \mathbf{J}\bar{\mathbf{Q}}_f + \mathbf{K}) \tag{S6b}$$

The projection of the vibrational eigenstates in the coordinate space corresponds to the vibrational wavefunctions. Focusing on the Rayleigh ($|\mathbf{v}_f^g\rangle = |\mathbf{0}^g\rangle$) and fundamental ($|\mathbf{v}_f^g\rangle = |\mathbf{0}^g + 1_k^g\rangle$, with $k = 1, N_{vib}$), the relevant wavefunctions are,

$$\langle\mathbf{0}^g + 1_k^g|\mathbf{Q}_i\rangle = \sqrt{2\Gamma_{ik}}Q_{ik}\frac{\det[\mathbf{\Gamma}_i]}{\pi^{N/4}}e^{-\frac{1}{2}\mathbf{Q}_i^t\mathbf{\Gamma}_i\mathbf{Q}_i} \tag{S7a}$$
$$\langle\mathbf{0}^g|\mathbf{Q}_i\rangle = \frac{\det[\mathbf{\Gamma}_i]}{\pi^{N/4}}e^{-\frac{1}{2}\mathbf{Q}_i^t\mathbf{\Gamma}_i\mathbf{Q}_i} \tag{S7b}$$
$$\langle\bar{\mathbf{Q}}_i|\mathbf{0}^g\rangle = \frac{\det[\mathbf{\Gamma}_i]}{\pi^{N/4}}e^{-\frac{1}{2}\bar{\mathbf{Q}}_i^t\mathbf{\Gamma}_i\bar{\mathbf{Q}}_i} \tag{S7c}$$

where $\mathbf{\Gamma}_i$ is a diagonal matrix with elements $\Gamma_{i,kk} = \omega_{i,k}/\hbar$.

Finally, we exploit the analytical expression for the off-diagonal matrix elements evaluated within the Path Integral formulation by Feynman,

$$\langle \mathbf{Q}_f | e^{-i\hat{H}_f \tau} | \bar{\mathbf{Q}}_f \rangle = \left(\frac{\det[\mathbf{a}_f(\tau)]}{(2\pi i\hbar)^N} \right)^{1/2} e^{\frac{i}{\hbar}\left[\frac{1}{2}\mathbf{Q}_f^t \mathbf{b}_f \mathbf{Q}_f + \frac{1}{2}\bar{\mathbf{Q}}_f^t \mathbf{b}_f \bar{\mathbf{Q}}_f - \mathbf{Q}_f^t \mathbf{a}_f \bar{\mathbf{Q}}_f\right]} \tag{S8}$$

where $\mathbf{a}$ and $\mathbf{b}$ are diagonal matrices, with elements,

$$(a_f(\tau))_{kk} = \frac{\omega_{f,k}}{\sin(\hbar\omega_{f,k}\tau)} \tag{S9a}$$

$$(b_f(\tau))_{kk} = \frac{\omega_{f,k}}{\tan(\hbar\omega_{f,k}\tau)} \tag{S9b}$$

Plugging the above expressions into Eq. S4, considering that $e^{iE_0 t/\hbar} = \frac{1}{\det[e^{-i\hbar\boldsymbol{\Gamma}_i}]^{1/2}}$, and after some manipulations (see Refs.[S3]), the correlation function can be expressed as,

$$\chi_{\rho\sigma} = \left(\frac{\det(\mathbf{a_f})\det(\mathbf{a_i}')}{(i\hbar)^{2N}} \right)^{1/2} e^{\frac{i}{\hbar}\mathbf{K}^t\mathbf{E}\mathbf{K}} \int_{-\infty}^{\infty}\int_{-\infty}^{\infty} d\mathbf{Q}_f d\bar{\mathbf{Q}}_f \, \mu_\rho^{gk}(\mathbf{Q}_f)\mu_\sigma^{gk}(\bar{\mathbf{Q}}_f) F_{v_f^g}(\mathbf{Q}_f) \exp\left[\frac{i}{\hbar}\left(\frac{1}{2}\mathbf{Q}_f^t\mathbf{B}\mathbf{Q}_f + \frac{1}{2}\bar{\mathbf{Q}}_f^t\mathbf{B}\bar{\mathbf{Q}}_f + \mathbf{K}^t\mathbf{E}\mathbf{J}(\mathbf{Q}_f + \bar{\mathbf{Q}}_f) - \mathbf{Q}_f^t\mathbf{A}\bar{\mathbf{Q}}_f\right)\right] \tag{S10}$$

where the factor $F_{v_f^g}(\mathbf{Q}_f)$ is 1 for the Rayleigh band and $\sqrt{2\Gamma_{ik}}(\boldsymbol{\alpha}\mathbf{Q}_f + K_k)$ for fundamental bands. We have introduced $\boldsymbol{\alpha}$ vector, with elements $\alpha_l \equiv J_{kl}$, and J_{kl} and K_k represent elements of the Duschinsky matrix $(\mathbf{J})$ and normal mode displacement vector $(\mathbf{K})$, respectively. Moreover,

the following matrices are introduced,

$$\mathbf{a_i'} = \frac{2i\hbar\boldsymbol{\Gamma}_i}{e^{-2i\hbar\boldsymbol{\Gamma}_i t}} \tag{S11a}$$

$$\mathbf{A} = \mathbf{a_f} \tag{S11b}$$

$$\mathbf{B} = \mathbf{b_f} - \mathbf{J}^t(i\hbar\boldsymbol{\Gamma}_i)\mathbf{J} \tag{S11c}$$

$$\mathbf{E} = i\hbar\boldsymbol{\Gamma}_i \tag{S11d}$$

The above Gaussian integral can be evaluated[S3] by first applying the change of variable,

$$\mathbf{Z} = \frac{1}{\sqrt{2}}(\mathbf{Q}_f + \bar{\mathbf{Q}}_f)\mathbf{U} = \frac{1}{\sqrt{2}}(\mathbf{Q}_f - \bar{\mathbf{Q}}_f) \tag{S12}$$

which decouples all coordinates in the integrand, followed by an additional change of variable to get Gaussian integrals in the standard form,

$$\mathbf{Z}_1 = \mathbf{D}^{1/2}\mathbf{Z} + \sqrt{2}\mathbf{D}^{-1/2}\mathbf{J}^t\mathbf{d}_i\mathbf{K} = \mathbf{D}^{1/2}\mathbf{Z} + \sqrt{2}\mathbf{D}^{-1/2}\boldsymbol{\lambda} \tag{S13a}$$

$$\mathbf{U}_1 = \mathbf{C}^{1/2}\mathbf{U} \tag{S13b}$$

where we have introduced the diagonal matrix $\mathbf{d}_i$ with elements $d_{i,kk} = \omega_{g,k}$ and the matrices,

$$\mathbf{C} = -\frac{i}{\hbar}(\mathbf{B} + \mathbf{A}) \tag{S14a}$$

$$\mathbf{D} = -\frac{i}{\hbar}(\mathbf{B} - \mathbf{A}) \tag{S14b}$$

(S14c)

After such transformations,[S3] the integral is simplified to,

$$\chi_{\rho\sigma} = \chi_0 \frac{1}{(2\pi)^N} \int_{-\infty}^{\infty} \int_{-\infty}^{\infty} d\mathbf{Z}_1 d\mathbf{U}_1 \exp\left[-\frac{1}{2}\mathbf{Z}_1^t\mathbf{Z}_1\right] \exp\left[-\frac{1}{2}\mathbf{U}_1^t\mathbf{U}_1\right] \\ \mu_\rho^{gk}(\mathbf{Z}_1,\mathbf{U}_1)\mu_\sigma^{gk}(\mathbf{Z}_1,\mathbf{U}_1)F_{v_f^g}(\mathbf{Z}_1,\mathbf{U}_1) \tag{S15}$$

where we have indicated that μ_ρ^{gk}, μ_σ^{gk} and $F_{v_f^g}$ are expressed in terms of the new variables, $\mathbf{Z}_1, \mathbf{U}_1$, and we grouped the common integral prefactor in χ_0, which reads,

$$\chi_0 = \sqrt{\frac{\det(\mathbf{a_f})\det(\mathbf{a_i}')}{(i\hbar)^{2N}\det(\mathbf{C})\det(\mathbf{D})}} \times \exp\left[-\mathbf{K}^T\mathbf{d_i}\mathbf{K} + \mathbf{K}^T\mathbf{d_i}\mathbf{J}\mathbf{D^{-1}}\mathbf{J^T}\mathbf{d_i}\mathbf{K}\right] \tag{S16}$$

The expressions for the transition dipoles in terms of the new coordinates, $\mathbf{Z}_1$, $\mathbf{U}_1$, read,

$$\mu_\rho^{gk}(\mathbf{Q}_f) = \mu_\rho^{gk,0} + (\boldsymbol{\mu}_\rho^{(1)})^t\mathbf{Q}_f = \mu_\rho^{gk,0} + \frac{\sqrt{2}}{2}(\boldsymbol{\mu}_\rho^{(1)})^t\mathbf{D}^{-1/2}\mathbf{Z}_1 + \frac{\sqrt{2}}{2}(\boldsymbol{\mu}_\rho^{(1)})^t\mathbf{C}^{-1/2}\mathbf{U}_1 + (\boldsymbol{\mu}_\rho^{(1)})^t\mathbf{D}_{HT} \tag{S17a}$$

$$\mu_\sigma^{kg}(\bar{\mathbf{Q}}_f) = \mu_\sigma^{kg,0} + (\boldsymbol{\mu}_\sigma^{(1)})^t\bar{\mathbf{Q}}_f = \mu_\sigma^{kg,0} + \frac{\sqrt{2}}{2}(\boldsymbol{\mu}_\sigma^{(1)})^t\mathbf{D}^{-1/2}\mathbf{Z}_1 - \frac{\sqrt{2}}{2}(\boldsymbol{\mu}_\sigma^{(1)})^t\mathbf{C}^{-1/2}\mathbf{U}_1 + (\boldsymbol{\mu}_\sigma^{(1)})^t\mathbf{D}_{HT} \tag{S17b}$$

where $\mathbf{D}_{HT} = -\mathbf{D}^{-1}\boldsymbol{\lambda}$, with $\boldsymbol{\lambda} = \mathbf{K}^t\mathbf{d}_i\mathbf{J}$. Multyplying both terms we arrive to the general expression,

$$\mu_\rho^{gk}\mu_\sigma^{kg} = \mathbf{Y}^0 + \mathbf{Y}^Z\mathbf{Z}_1 + \mathbf{Y}^U\mathbf{U}_1 + \mathbf{Z}_1^t\mathbf{Y}^{ZZ}\mathbf{Z}_1 + \mathbf{U}_1^t\mathbf{Y}^{UU}\mathbf{U}_1 + \mathbf{Z}_1^t\mathbf{Y}^{ZU}\mathbf{U}_1 \tag{S18}$$

with,

$$\mathbf{Y}^0 = \mu_\rho^{gk,0}\mu_\sigma^{kg,0} + (\mu_\rho^{gk,0}(\boldsymbol{\mu}_\sigma^{(1)})^t + \mu_\sigma^{kg,0}(\boldsymbol{\mu}_\rho^{(1)})^t)\mathbf{D}_{HT} + \mathrm{Tr}[\mathbf{M}_{\rho\sigma}\mathbf{D}_{HT}\mathbf{D}_{HT}^t] \tag{S19a}$$

$$\mathbf{Y}^Z = \frac{\sqrt{2}}{2}\left[\mu_\rho^{gk,0}(\boldsymbol{\mu}_\sigma^{(1)})^t + \mu_\sigma^{kg,0}(\boldsymbol{\mu}_\rho^{(1)})^t + \mathbf{D}_{HT}(\mathbf{M}_{\sigma\rho} + \mathbf{M}_{\rho\sigma})\right]\mathbf{D}^{-1/2} \tag{S19b}$$

$$\mathbf{Y}^U = \frac{\sqrt{2}}{2}\left[\mu_\rho^{gk,0}(\boldsymbol{\mu}_\sigma^{(1)})^t + \mu_\sigma^{kg,0}(\boldsymbol{\mu}_\rho^{(1)})^t - \mathbf{D}_{HT}(\mathbf{M}_{\sigma\rho} - \mathbf{M}_{\rho\sigma})\right]\mathbf{C}^{-1/2} \tag{S19c}$$

$$\mathbf{Y}^{ZZ} = \frac{1}{2}\mathbf{D}^{-1/2}\mathbf{M}_{\rho\sigma}\mathbf{D}^{-1/2} \tag{S19d}$$

$$\mathbf{Y}^{UU} = -\frac{1}{2}\mathbf{C}^{-1/2}\mathbf{M}_{\rho\sigma}\mathbf{C}^{-1/2} \tag{S19e}$$

$$\mathbf{Y}^{ZU} = \frac{1}{2}\mathbf{D}^{-1/2}(\mathbf{M}_{\sigma\rho} - \mathbf{M}_{\rho\sigma})\mathbf{C}^{-1/2} \tag{S19f}$$

where we have introduced the matrix $\mathbf{M}_{\sigma\rho}$ with elements $(M_{\sigma\rho})_{ij} = (\mu_\sigma^{(1)})_i(\mu_\rho^{(1)})_j$.

Before integrating, we also need to specify the type of bands (Rayleigh or fundamentals). Each type is revised in the following subsections.

S1.1.1 Rayleigh band

For the Rayleigh band, $F_{v_f^g} = 1$, and the correlation functions reads,

$$\begin{aligned}\chi_{\rho\sigma}^{|\mathbf{0}^g\rangle} &= \chi_0\frac{1}{(2\pi)^N}\int_{-\infty}^{\infty}\int_{-\infty}^{\infty} d\mathbf{Z}_1 d\mathbf{U}_1 \exp\left[-\frac{1}{2}\mathbf{Z}_1^t\mathbf{Z}_1\right]\exp\left[-\frac{1}{2}\mathbf{U}_1^t\mathbf{U}_1\right] \\ &\qquad \left(\mathbf{Y}^0 + \mathbf{Y}^Z\mathbf{Z}_1 + \mathbf{Y}^U\mathbf{U}_1 + \mathbf{Z}_1^t\mathbf{Y}^{ZZ}\mathbf{Z}_1 + \mathbf{U}_1^t\mathbf{Y}^{UU}\mathbf{U}_1 + \mathbf{Z}_1^t\mathbf{Y}^{ZU}\mathbf{U}_1\right) \\ &= \chi_0\frac{1}{(2\pi)^N}\int_{-\infty}^{\infty}\int_{-\infty}^{\infty} d\mathbf{Z}_1 d\mathbf{U}_1 \exp\left[-\frac{1}{2}\mathbf{Z}_1^t\mathbf{Z}_1\right]\exp\left[-\frac{1}{2}\mathbf{U}_1^t\mathbf{U}_1\right] \\ &\qquad \left(\mathbf{Y}^0 + \mathbf{Z}_1^t\mathbf{Y}^{ZZ}\mathbf{Z}_1 + \mathbf{U}_1^t\mathbf{Y}^{UU}\mathbf{U}_1\right)\end{aligned} \tag{S20}$$

where we take into account that only integrals with even exponents on $Z_{1,i}$ and $U_{1,i}$ are non-vanishing. Further using the expression for Gaussian integrals,

$$\int_{-\infty}^{\infty} dx\ e^{x^2/2} = \sqrt{2\pi} \tag{S21a}$$

$$\int_{-\infty}^{\infty} dx\ x^n e^{x^2/2} = (1 \cdot 3 \cdots (n-1))\sqrt{2\pi} \quad (n > 0 \text{ and even}) \tag{S21b}$$

the above integral can be fully evaluated leading to,

$$\begin{aligned} \chi_{\rho\sigma}^{|\mathbf{0}^g\rangle}(t,T) = \ & \chi_0 \Bigg[\mu_\rho^{gm,(0)} \mu_\sigma^{gm,(0)} + \mu_\rho^{gm,(0)} \sum_{i=1}^{N_{vib}} \mu_{\sigma,i}^{gm,(1)} (D_{HT})_i + \\ & \mu_\sigma^{gm,(0)} \sum_i \mu_{\rho,i}^{gm,(1)} (D_{HT})_i + \sum_{i,j} \mu_{\rho,i}^{gm,(1)} \mu_{\sigma,j}^{gm,(1)} (A_{HT})_{ji} \Bigg] \end{aligned} \tag{S22}$$

where the sums run over the N_{vib} normal mode coordinates and we introduce the $\mathbf{A}_{HT}$ matrix as,

$$\mathbf{A}_{HT} = \mathbf{D}_{HT}\mathbf{D}_{HT}^t + \frac{1}{2}(\mathbf{D}^{-1} - \mathbf{C}^{-1}) \tag{S23}$$

S1.1.2 Fundamental bands

In the case of the k-th fundamental,

$$F_{v_f^g}(\mathbf{Q}_f) = \sqrt{2\Gamma_{ik}}(\boldsymbol{\alpha}\mathbf{Q}_f + K_k) = \sqrt{2\Gamma_{ik}} \left(\frac{\sqrt{2}}{2}\boldsymbol{\alpha}^t\mathbf{D}^{-1/2}\mathbf{Z}_1 + \frac{\sqrt{2}}{2}\boldsymbol{\alpha}^t\mathbf{C}^{-1/2}\mathbf{U}_1 + \boldsymbol{\alpha}^t\mathbf{D}_{HT} + K_k \right) \tag{S24}$$

and the correlation function reads,

$$\chi_{\rho\sigma}^{|\mathbf{0}^g+1_k\rangle} = \sqrt{2\Gamma_{ik}}\chi_0 \frac{1}{(2\pi)^N} \int_{-\infty}^{\infty}\int_{-\infty}^{\infty} d\mathbf{Z}_1 d\mathbf{U}_1 \exp\left[-\frac{1}{2}\mathbf{Z}_1^t\mathbf{Z}_1\right] \exp\left[-\frac{1}{2}\mathbf{U}_1^t\mathbf{U}_1\right]$$
$$\left(\frac{\sqrt{2}}{2}\boldsymbol{\alpha}^t\mathbf{D}^{-1/2}\mathbf{Z}_1 + \frac{\sqrt{2}}{2}\boldsymbol{\alpha}^t\mathbf{C}^{-1/2}\mathbf{U}_1 + \boldsymbol{\alpha}^t\mathbf{D}_{HT} + K_k\right) \tag{S25}$$
$$\left(\mathbf{Y}^0 + \mathbf{Y}^Z\mathbf{Z}_1 + \mathbf{Y}^U\mathbf{U}_1 + \mathbf{Z}_1^t\mathbf{Y}^{ZZ}\mathbf{Z}_1 + \mathbf{U}_1^t\mathbf{Y}^{UU}\mathbf{U}_1 + \mathbf{Z}_1^t\mathbf{Y}^{ZU}\mathbf{U}_1\right)$$

again, we take into account that only integrals with even exponents on $Z_{1,i}$ and $U_{1,i}$ are non-vanishing. Further operating all the resulting Gaussian, we arrive to,

$$\chi_{\rho\sigma}^{|\mathbf{0}^g+1_k\rangle}(t,T) = \chi_0\sqrt{2\Gamma_{gk}}\Bigg[\mu_\rho^{gm,(0)}\mu_\sigma^{gm,(0)}\left(K_k - \sum_i J_{ki}(D_{HT})_i\right) +$$
$$\mu_\rho^{gm,(0)}\left(K_k\sum_i \mu_{\sigma,i}^{gm,(1)}(D_{HT})_i - \sum_{i,j} J_{ki}\mu_{\sigma,j}^{gm,(1)}\left[(D_{HT})_i(D_{HT})_j - \frac{1}{2}((D^{-1})_{ji} - (C^{-1})_{ji})\right]\right) +$$
$$\mu_\sigma^{gm,(0)}\left(K_k\sum_i \mu_{\rho,i}^{gm,(1)}(D_{HT})_i - \sum_{i,j} J_{ki}\mu_{\rho,j}^{gm,(1)}\left[(D_{HT})_i(D_{HT})_j - \frac{1}{2}((D^{-1})_{ji} + (C^{-1})_{ji})\right]\right) +$$
$$\sum_{ij}\mu_{\rho,i}^{gm,(1)}\mu_{\sigma,j}^{gm,(1)}\Bigg(K_k(A_{HT})_{ji} - (A_{HT})_{ji}\sum_l J_{kl}(D_{HT})_l +$$
$$\frac{1}{2}(D_{HT})_j\sum_l J_{kl}((D^{-1})_{il} + (C^{-1})_{il}) + \frac{1}{2}(D_{HT})_i\sum_l J_{kl}((D^{-1})_{jl} - (C^{-1})_{jl})\Bigg)\Bigg] \tag{S26}$$

We note that the analytical expression for overtones and combination bands can also be derived following similar steps. The resulting expression involve, though, a considerable increased computational cost, mainly at HT level. The computational cost can be reduced adopting some approximations.[S4]

S2 An example of the full 2D nonadiabatic vibrational RR spectra computed with our protocol

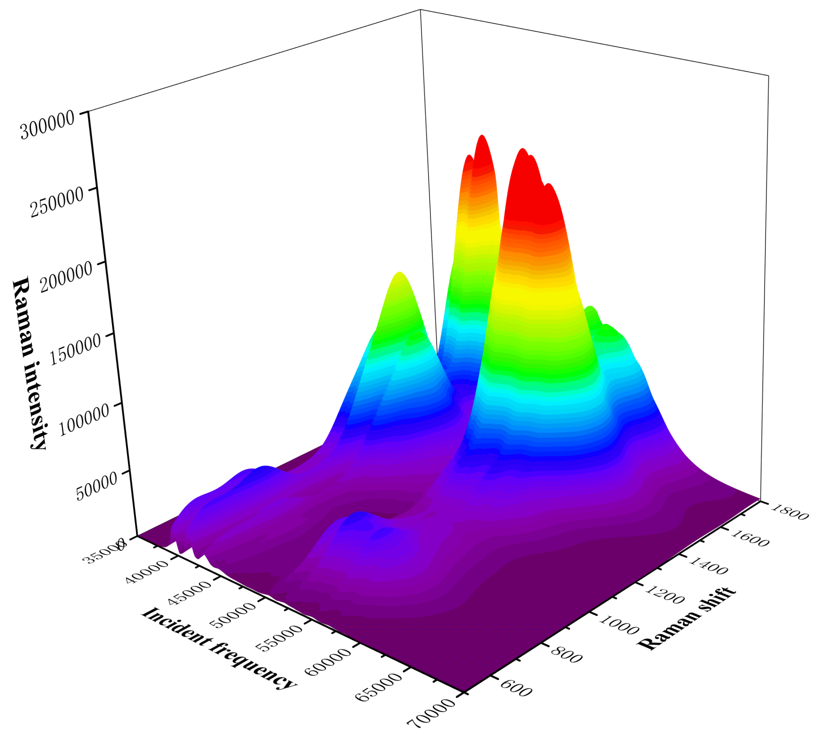

Figure S1: 2D vibrational Resonance Raman spectrum of Thymine with the predictions of LVC model considering the first seven excited states parameterized with CAM-B3LYP/6-311G+(d,p) calculations in gas phase. The damping γ is 0.04 eV and the broadening along the Raman shift is a Lorentzian with HWHM 15 cm^{-1}

S3 Technical checks

S3.1 FCclasses and ML-MCTDH deliver equivalent results for "single-state" cases

In this section we show that for cases in which the inter-state couplings are set to zero ("single state"), computations performed with analytical correlation functions by FCclasses3[S2] and with numerical wavepacket ML-MCTDH propagations by Quantics deliver practically indistinguishable results.

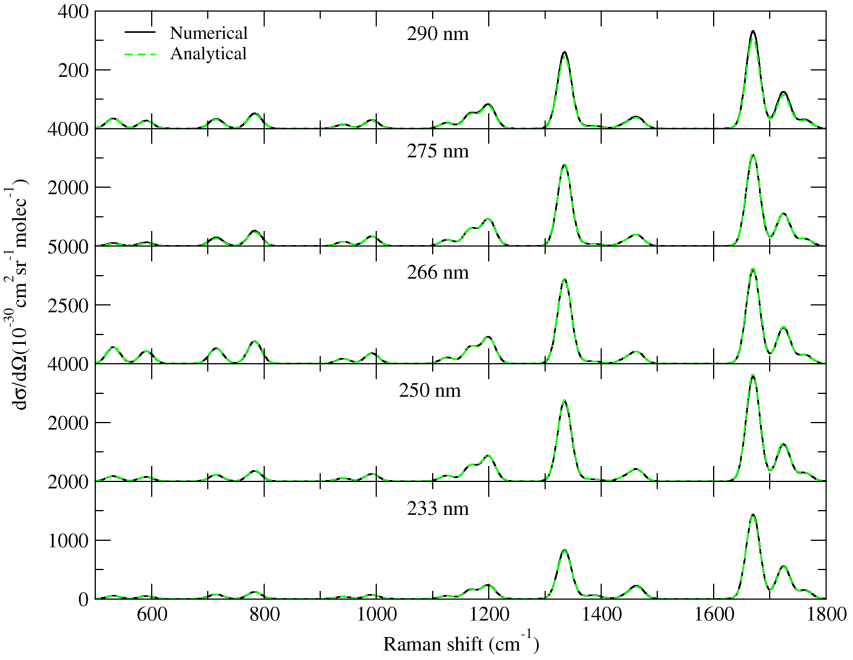

Figure S2: Comparison of the computations of the vibrational Resonance Raman spectrum of Thymine with the model FC|VG Int (i.e. including interferences) obtained either with FC-classes and analytical correlation functions ("analytical") or with numerical propagations with ML-MCTDH ("numerical") with the LVC model setting the inter-state couplings to zero. Computations including the effect of the first seven states on the grounds of CAM-B3LYP/6-311G+(d,p) computations in gas-phase, with a damping γ = 0.04 eV.

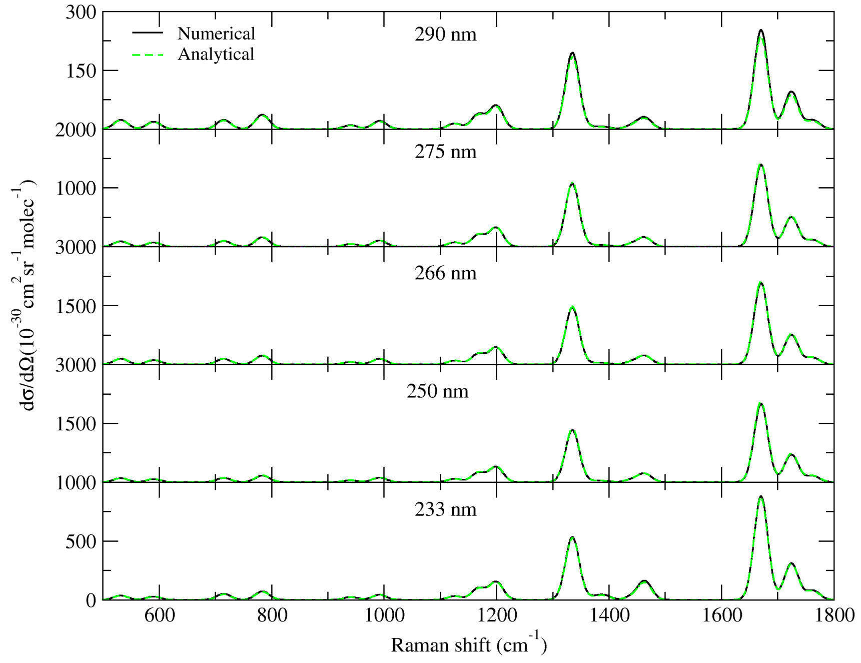

Figure S3: Comparison of the computations of the vibrational Resonance Raman spectrum of Thymine with the model FC|VG Int (i.e. including interferences) obtained either with FC-classes and analytical correlation functions ("analytical") or with numerical propagations with ML-MCTDH ("numerical") with the LVC model setting the inter-state couplings to zero. Computations including the effect of the first seven states on the grounds of CAM-B3LYP/6-311G+(d,p) computations in gas-phase, with a damping $\gamma = 0.12$ eV.

S4 Additional results in gas phase

S4.1 Effect of the basis set on the absorption spectrum.

Table S1: Symmetry, vertical excitation energies E_{gf} (eV), oscillator strengths (δ_{OPA}) of the first seven excited states for Thymine, calculated with CAM-B3LYP and the 6-31G(d) and 6-311G+(d,p) basis sets in gas phase.

STATE	6-31G(d)					
	Sym.	E_{gf}(eV)	δ_{OPA}	Trans.	Char.	Coeff.
S_1	A''	5.15	0.0001	H-1→L	$n_O\pi_1^*$	0.63
S_2	A'	5.51	0.18	H→L	$\pi\pi_1^*$	0.69
S_3	A''	6.48	0.00	H-1→L+1	$n_O\pi_2^*$	0.44
				H-3→L+1		0.44
S_4	A'	6.83	0.077	H-2→L	$\pi\pi_2^*$	0.69
S_5	A'	6.96	0.17	H→L+1	$\pi\pi_3^*$	0.69
S_6	A''	7.23	0.00	H-3→L	$n_O\pi_3^*$	0.62
S_7	A''	7.49	0.00	H→L+2	$\pi Ry_{\sigma 1}$	0.69
STATE	6-311G+(d,p)					
	Sym.	E_{gf}(eV)	δ_{OPA}	Trans.	Char.	Coeff.
S_1	A''	5.14	0.00	H-1→L	$n_O\pi_1^*$	0.63
S_2	A'	5.31	0.19	H→L	$\pi\pi_1^*$	0.69
S_3	A''	5.94	0.0006	H→L+1	$\pi Ry_{\sigma 1}$	0.69
S_4	A''	6.47	0.00	H-1→L+4	$n_O\pi_2^*$	0.40
				H-3→L+4		0.38
S_5	A'	6.67	0.055	H-2→L	$\pi\pi_2^*$	0.69
S_6	A'	6.73	0.22	H→L+4	$\pi\pi_3^*$	0.66
S_7	A''	6.78	0.0013	H→L+3	$\pi Ry_{\sigma 2}$	0.60

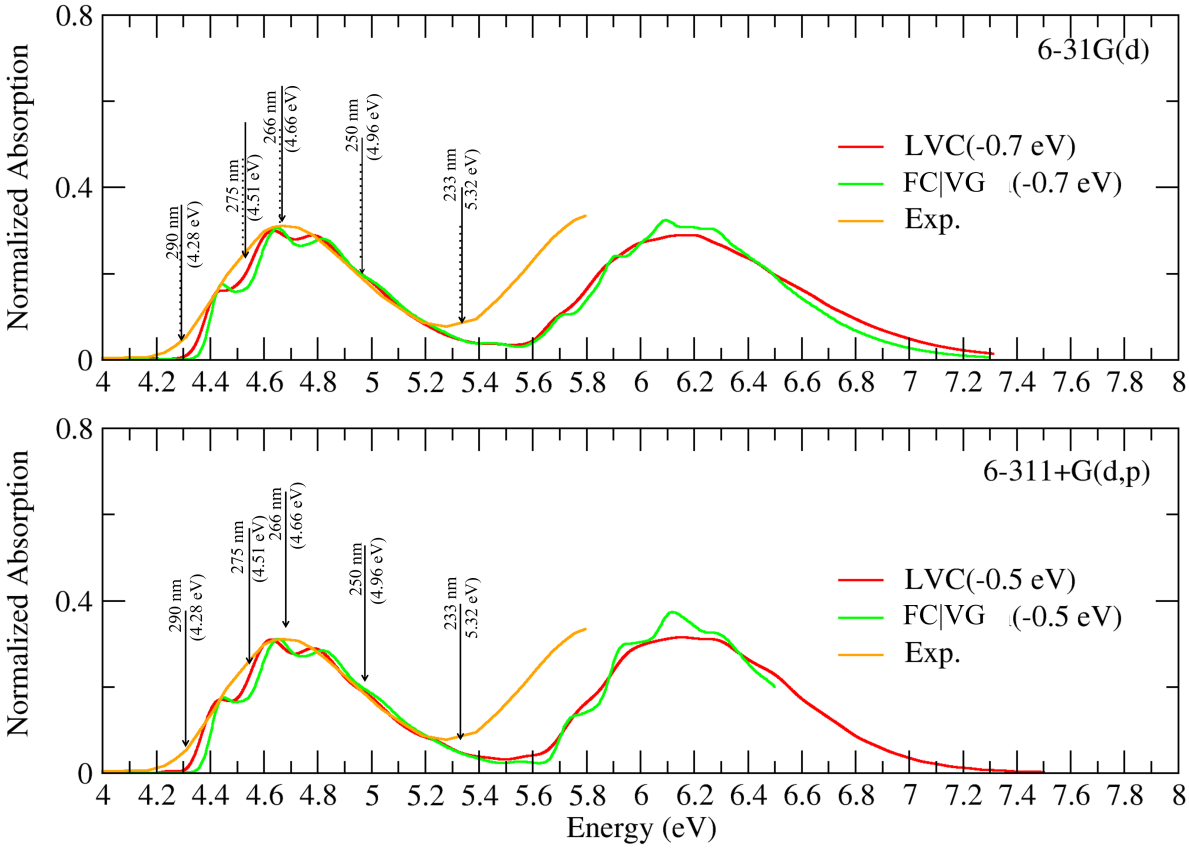

Figure S4: Absorption spectra of Thymine with the predictions of FC|VG and the LVC model, convoluted with a Gaussian of HWHM = 0.04 eV. Experimental data[S5] is in aqueous solution.

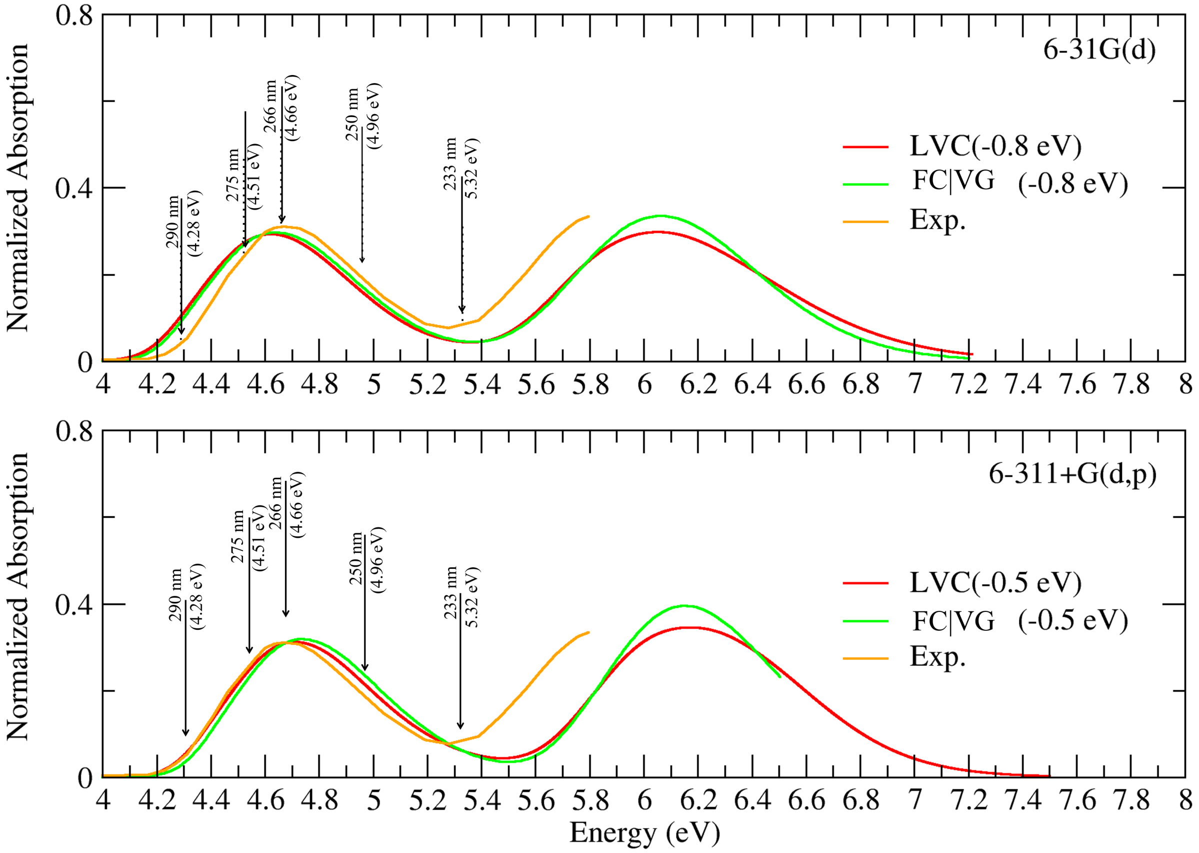

Figure S5: Absorption spectra of Thymine with the predictions of FC|VG and the LVC model, convoluted with a Gaussian of HWHM = 0.12 eV.Experimental data[S5] is in aqueous solution.

S4.2 Effect of the broadening along ω_I on the vRR spectra

Results obtained with damping (a broadening along ω_I) with γ=0.04 eV or 0.12 eV are quite similar, apart from a general smoothing of the Raman excitation profiles (right panels, see also Figure S11) for γ=0.12 eV, which translates into a general decrease (or an hard-to-detect increase for ω_I out of resonance) of the intensity in 1D vRR spectra (left panels) as a function of the Raman shift.

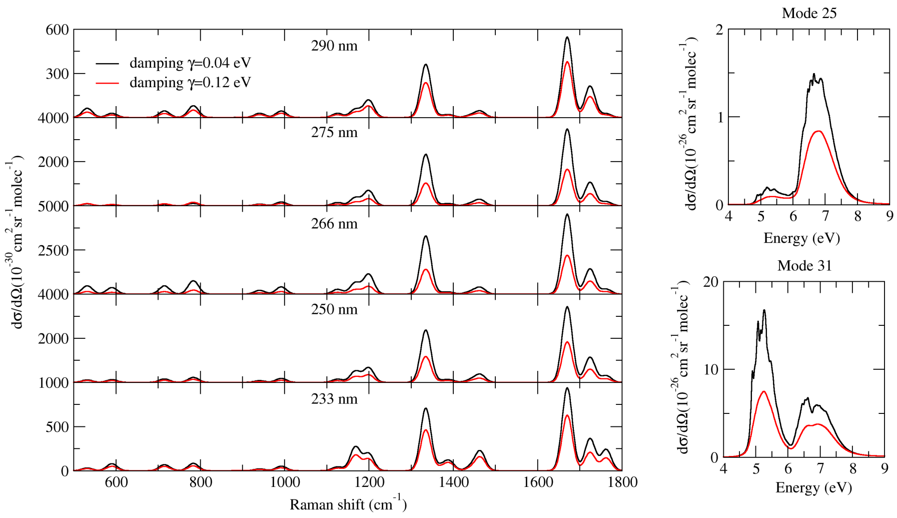

Figure S6: Vibrational resonance Raman spectra of Thymine with the predictions of LVC model considering the first 7 excited states with two Raman excitation profiles, computed with damping γ = 0.04 eV and 0.12 eV.

S4.3 Comparing LVC results with 3 and 7 states

Figure S7 shows that up to 250 nm the results of LVC models comprising the lowest 3 or 7 states are virtually identical. Among the three lowest states just $\pi\pi^*_1$ is bright and it is dominating the vRR signal.

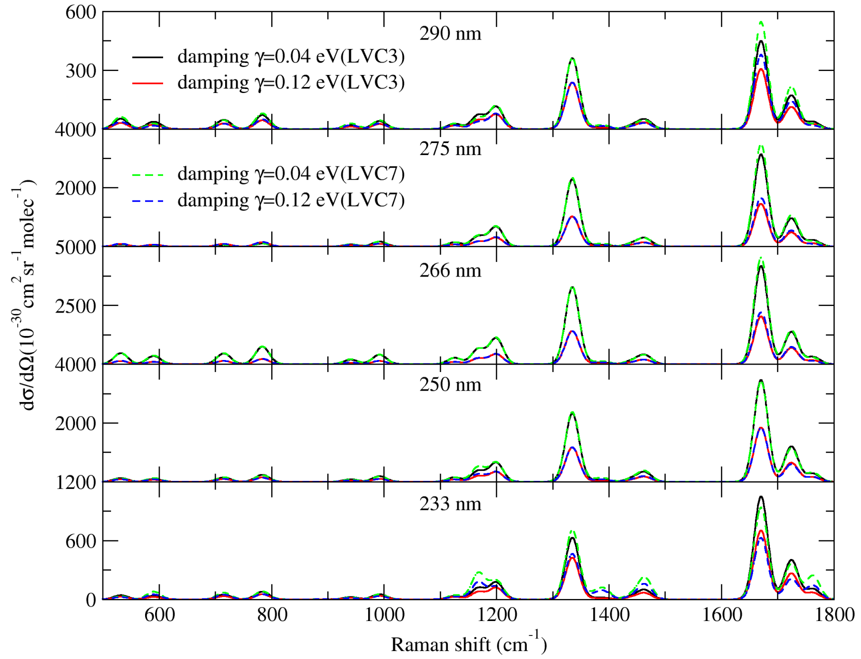

Figure S7: Vibrational resonance Raman spectra of Thymine with the predictions of LVC model, computed with damping $\gamma = 0.04$ eV and 0.12 eV.

S4.4 Single-state approaches for $\pi\pi_1^*$

S4.4.1 Effect of different PES models. Predictions of VH and AH models for $\pi\pi_1^*$

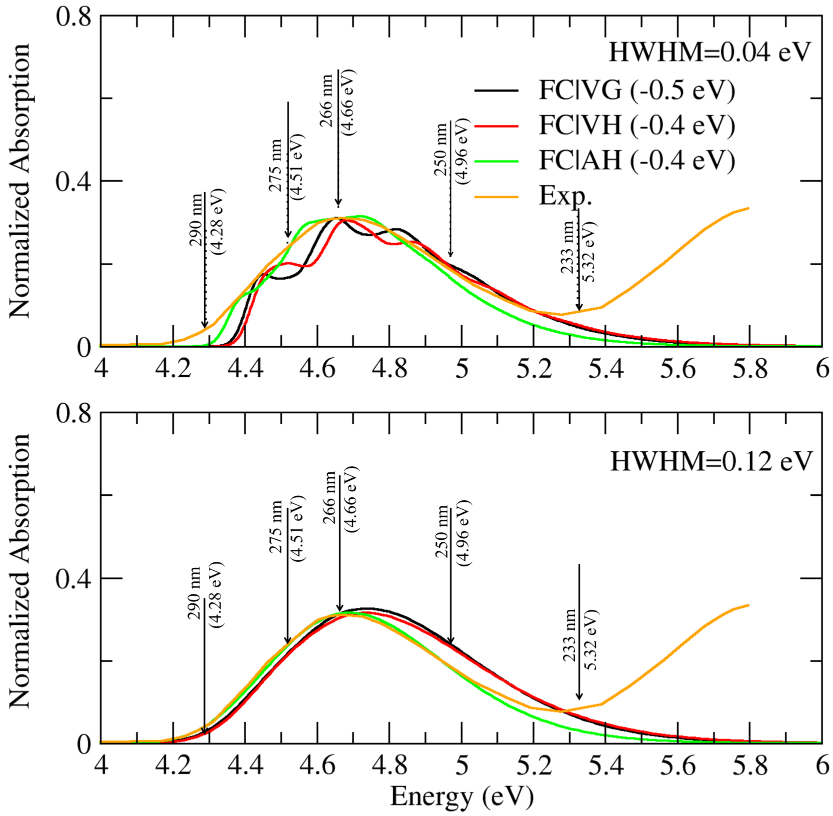

Figure S8: Absorption spectra of Thymine with the predictions of FC|VG, FC|VH, and FC|AH for the first $\pi\pi_1^*$, convoluted with a Gaussian of HWHM = 0.04 eV and HWHM = 0.12 eV. Experimental data[S5] is in aqueous solution

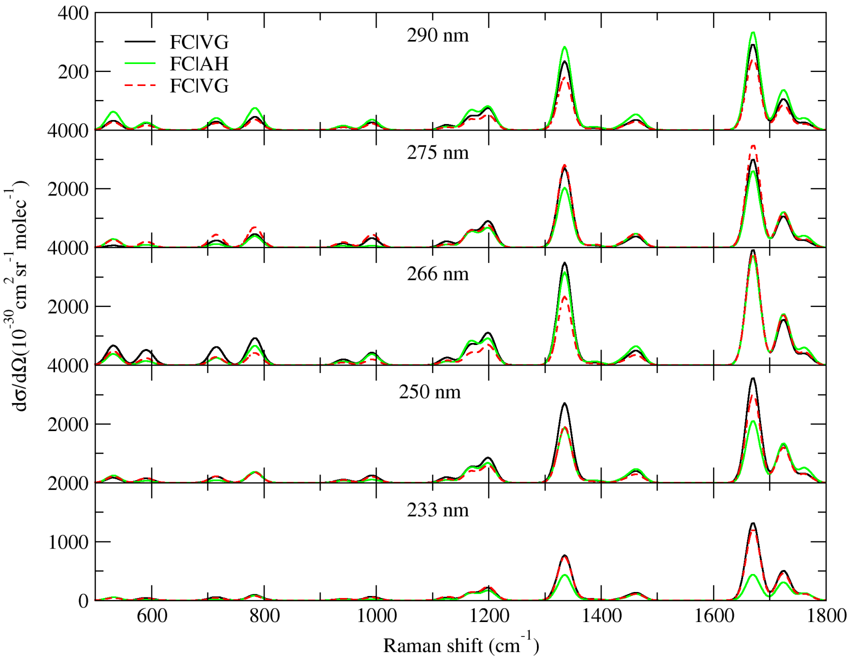

Figure S9: Vibrational resonance Raman spectra of Thymine with the predictions of FC|VG, FC|VH and FC|AH model, obtained in CAM-B3LYP/6-311G+(d,p), computed with damping γ = 0.04 eV. To simulate the experimental resonance conditions, the excitation frequencies have been blue-shifted by 0.4 eV for AH and VH and by 0.5 eV for VG (see absorption spectra in Figure S8). Here and in the following figure, with the VH model we predict 3 imaginary frequency modes on $\pi\pi_1^*$: 1 (377.5450i), 2 (210.2680i), and 3 (154.0397i), whereas there is no imaginary frequency for AH model. For VH calculations imaginary modes were simply turned to real. Of course this is an arbitrary choice and it should be necessary to check that it is not affecting the results. This has not been done since VH results are very similar to AH and VG ones. On one side, this finding provides an indirect support to their reliability, on the other side it makes them not very interesting, so that they will not be discussed further.

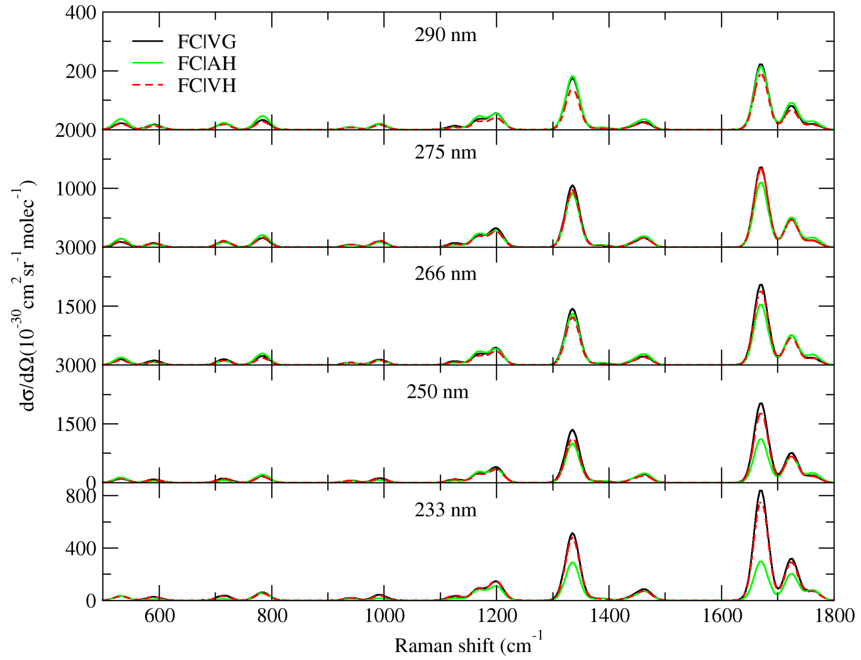

Figure S10: Vibrational resonance Raman spectra of Thymine with the predictions of FC|VG,FC|VH and FC|AH model ,obtained in CAM-B3LYP/6-311G+(d,p), computed with damping $\gamma = 0.12$ eV. To simulate the experimental resonance conditions, the excitation frequencies have been blue-shifted by 0.4 eV for AH and VH and by 0.5 eV for VG (see absorption spectra in Figure S8).

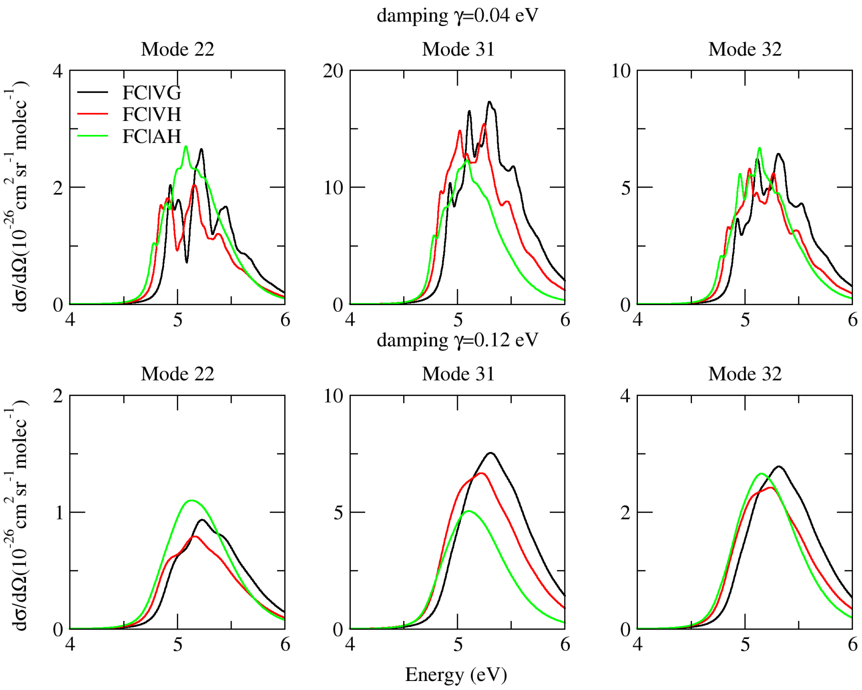

Figure S11: Raman excitation profiles of the three modes of Thymine from FC|VG, FC|VH, FC|AH model for $\pi\pi_1^*$. All spectra computed with damping $\gamma = 0.04$ eV (top) and 0.12 eV (bottom), obtained with CAM-B3LYP/6-311G+(d,p).

S4.5 "Single-state" analysis of the effect of the $\pi\pi_2^*$ and $\pi\pi_3^*$ on the vRR spectrum

Figure S12 reports the contribution predicted for each of the three bright $\pi\pi^*$ states with single-state FC|VG approaches at an excitation energy corresponding to 233 nm in the experiment. It shows that both $\pi\pi_2^*$ and $\pi\pi_3^*$ predict new bands in the region 1400-1500 cm^{-1} which however are too weak with respect to those arising from $\pi\pi_1^*$. Figure S13 shows that these new bands become more intense (especially those due to $\pi\pi_3^*$) if we artificially red-shift $\pi\pi_2^*$ and $\pi\pi_3^*$ by 0.3 eV with respect to $\pi\pi_1^*$, as suggested by the comparison between the experimental and computed absorption bands.

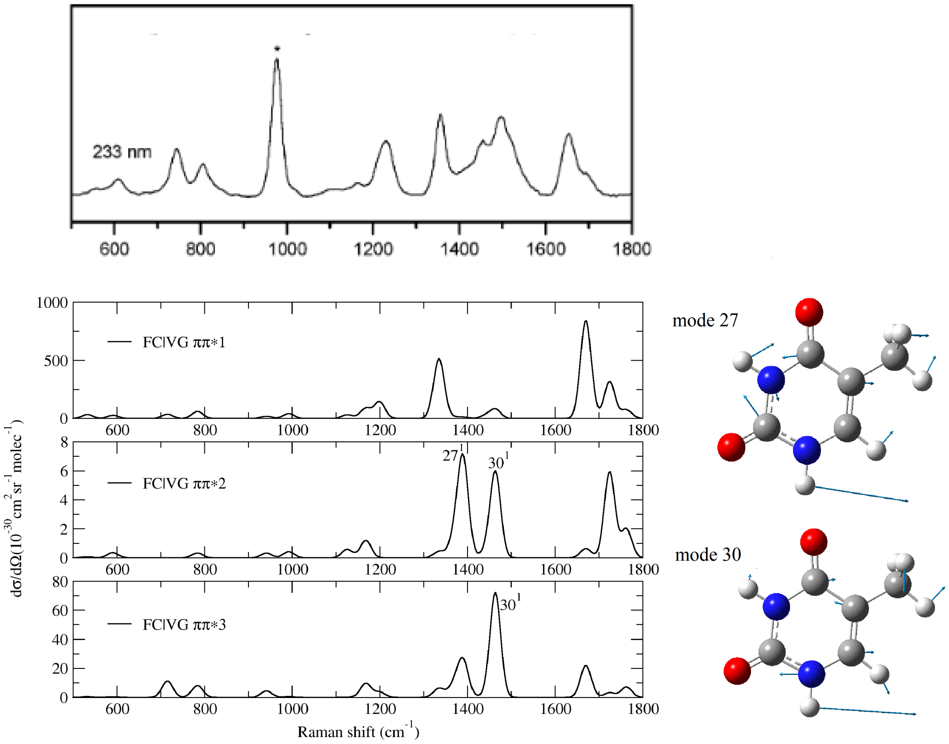

Figure S12: Vibrational resonance Raman spectra of Thymine with the predictions of FC|VG model, excited at 5.32 eV (233 nm), obtained with CAM-B3LYP/6-311G+(d,p), computed with damping $\gamma = 0.12$ eV. The experimental data reported as insets is from[S5] in aqueous solutions.

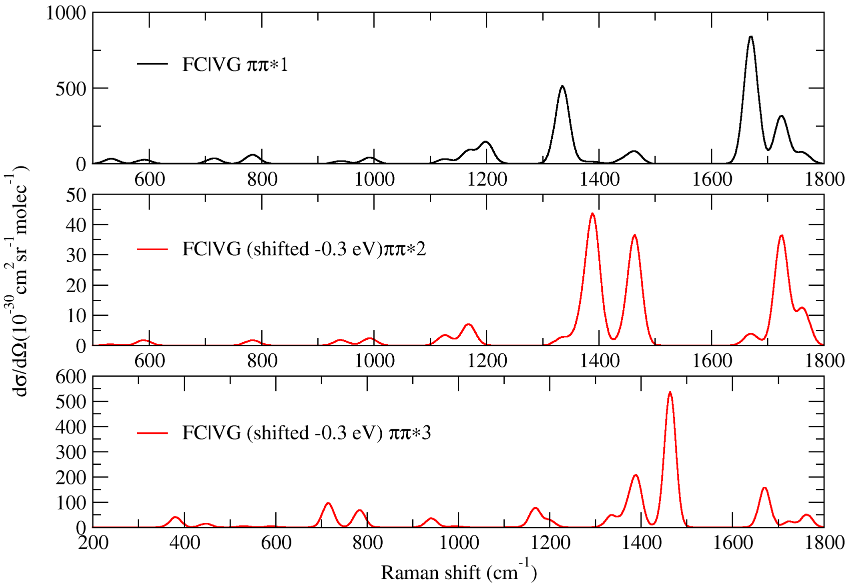

Figure S13: Vibrational resonance Raman spectra of Thymine with the predictions of FC|VG model, excited at 5.32 eV (233 nm), obtained with CAM-B3LYP/6-311G+(d,p), computed with damping $\gamma = 0.12$ eV. The energy of $\pi\pi^*_2$ and $\pi\pi^*_2$ are artificially red-shifted by 0.3 eV

S4.6 LVC Raman excitation profiles for the six modes studied in the main text, without shifting $\pi\pi_2^*$ and $\pi\pi_3^*$

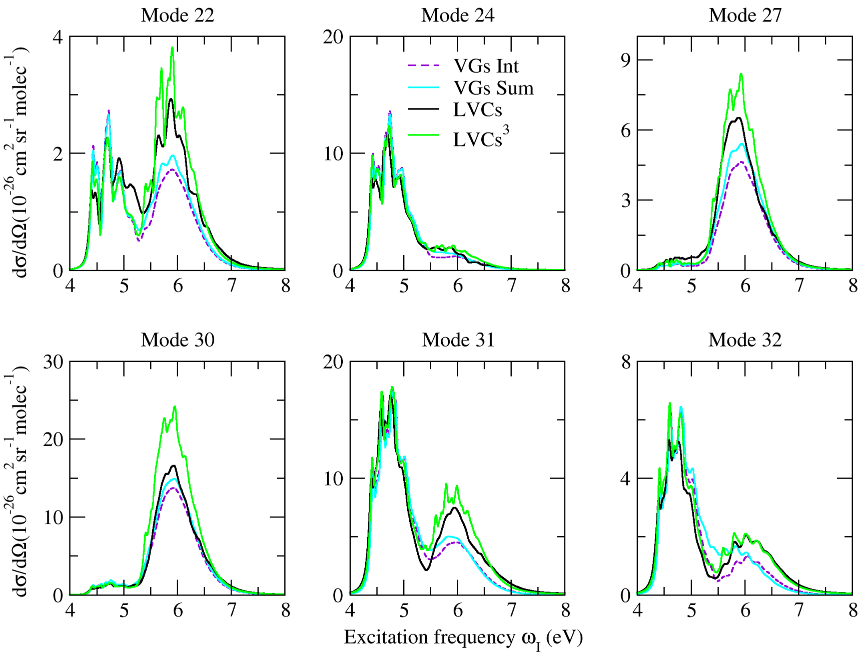

Figure S14: Raman excitation profile of six modes of Thymine with the first seven excited states with the $\pi\pi_2^*$ and $\pi\pi_3^*$ artificially red-shifted by 0.3 eV (LVC$_S$), FC|VG$_S$ Int , FC|VG$_S$ Sum and LVC$_S^3$ (only considering the contribution of the three lowest $\pi\pi^*$) obtained in CAM-B3LYP/6-311G+(d,p), applying a Lorentzian damping with $\gamma = 0.04$ eV. The excitation frequencies adopted in the computations to reproduce the experimental resonance conditions are here red-shifted by 0.5 eV. In this way they matches the experimental ones.

Figure S14 report the Raman excitation profiles obtained with LVC, VG Int and VG Sum models for six modes in Figure 6 where $\pi\pi^*2$ and $\pi\pi^*3$ have been red-shifted by 0.3 eV with respect to the lowest states and compare them with a different LVC model (LVC$_S^3$) in which we consider the coupling of only the three bright states (i.e. only those considered in VG models). Green and black lines show significant changes indicating that also the coupling with darks states play a role. Very interestingly however with respect to LVC, LVC$_S^3$ results are not closer to the ones obtained with VG models and the same states, confirming that even using the same set of states VG Int cannot reproduce reliably the effect of the quasi-resonance of the electronic states.

The results of Figure S15 report again the same Raman excitation profiles, but here however $\pi\pi^*2$ and $\pi\pi^*3$ have not been red-shifted by 0.3 eV for reproducing the energy gaps deduced from the analysis of the absorption spectra. Very interestingly computations without this shift (to be compared with those shifted in Figure 6), predict quite different effects of the inter-state couplings. In particular for the second band at $\sim$ 6.0 eV it is predicted that inter-state couplings lead to a decrease of the Raman intensity while the opposite occurs when the shift is applied (see Figure 6 in the main text or Figure S14).

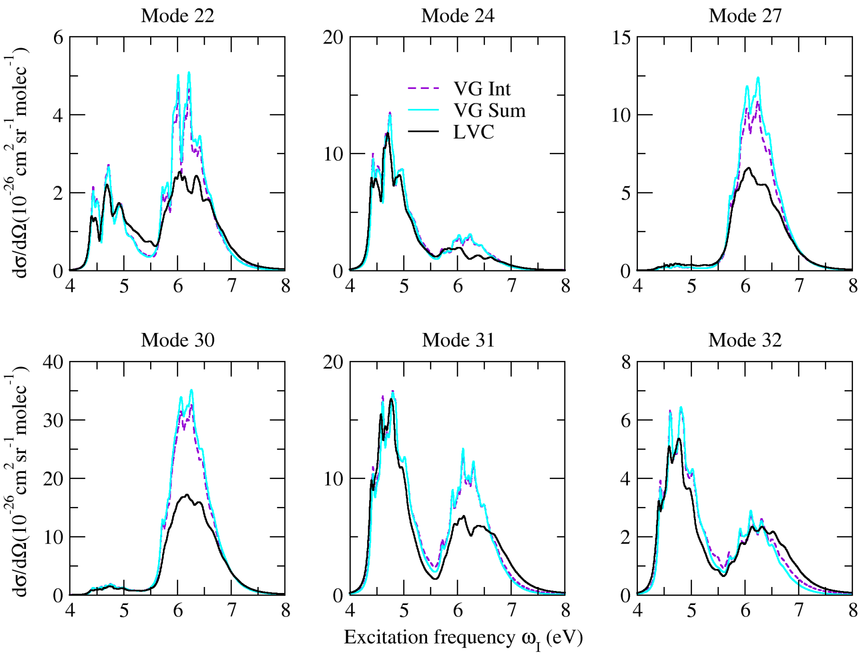

Figure S15: Raman excitation profiles of the six modes of Thymine, computed at non-adiabatic LVC level, and with VG Int and VG Sum models, obtained in Cam-B3LYP/6-311G+(d,p), computed with damping $\gamma = 0.04$ eV. The excitation frequencies adopted in the computations to reproduce the experimental conditions are here red-shifted by 0.5 eV so to match the experimental ones.

S4.7 LVC Raman excitation profiles for three modes observed in the experiment

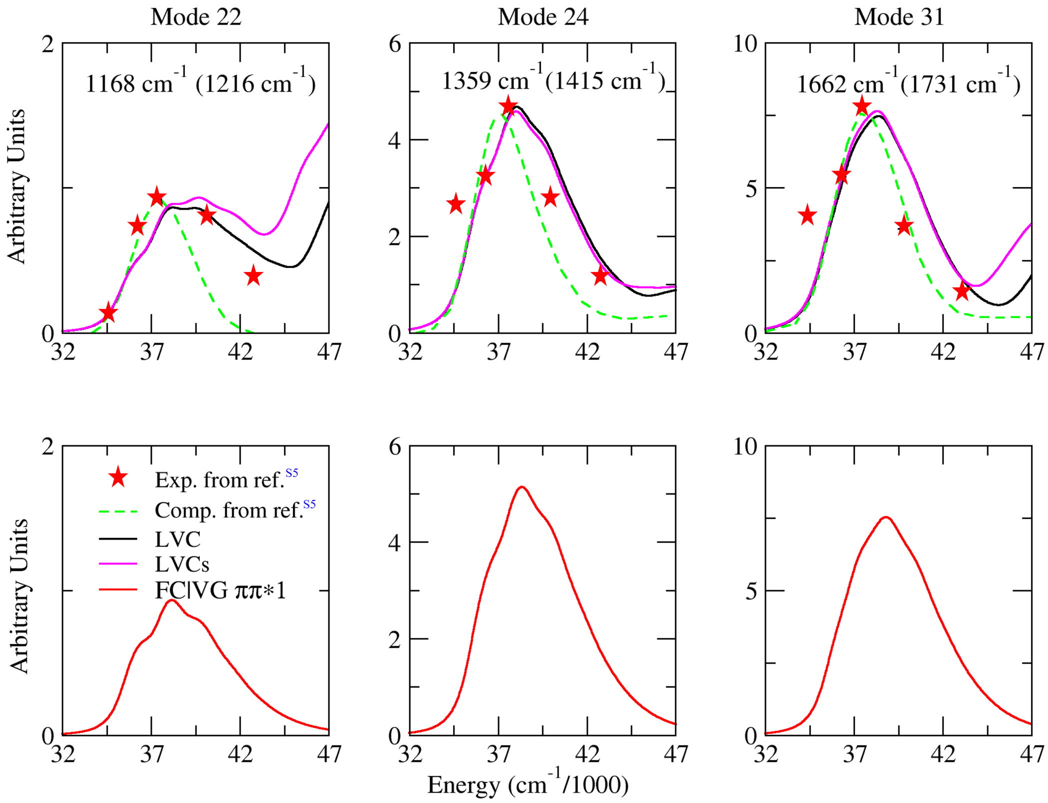

Figure S16: Raman excitation profiles of the three modes of Thymine, computed at non-adiabatic LVC level, and with LVCs and FC|VG models, obtained in Cam-B3LYP/6-311G+(d,p), computed with damping $\gamma = 0.12$ eV. Experimental frequencies are out of the parentheses, our computational frequencies are in the parentheses. Intensity is renormalized and our computational wavelength are blue-shifted by 4032 cm^{-1} (0.5 eV) to match experiment.

Figure S16 compares the Raman excitation profiles computed for 3 selected modes with the LVC model and with the LVCs model adopted in the main text where the energy gap between $\pi\pi^*1$ and the higher states $\pi\pi^*2$ and $\pi\pi^*3$ has been set to a value more similar to experiment, with those obtained considering only the $\pi\pi^*1$ contribution at FC|VG level, with the computations (equivalent to VG) performed in ref.[S5] considering only $\pi\pi^*1$ and, finally, with the experimental points measured in the same ref.[S5] It can be seen that our profiles become more asymmetric

when considering simultaneously the contributions of the three states and their couplings. As a consequence, LVC results seem in better agreement with experiment than VG ones.

S4.8 Further tests on Herzberg-Teller effects

S4.8.1 The contribution of the two lowest excited dark states

Figures S17 and S18 show the "single-state" computation of the vRR of the two lowest-energy and almost dark states n$_O\pi^*$ and $\pi Ry_\sigma 1$ respectively, at the different excitation wavelengths with FC|VG and FCHT|VG level of theory. They show that HT effects dominate the FC ones since FCHT spectra are more intense than FC ones by respectively a factor 10^6 (n$_O\pi^*$) and 10^3 ($\pi Ry_\sigma 1$). By comparing these results with those for $\pi\pi^*1$ in Figure S9 one can notice that, despite the huge enhancement, the contributions of these two quasi-dark states are predicted to remain much smaller than those of $\pi\pi^*1$, which, in fact, dominates the spectrum (check the full LVC prediction with 7 states in Figure 4 in the main text).

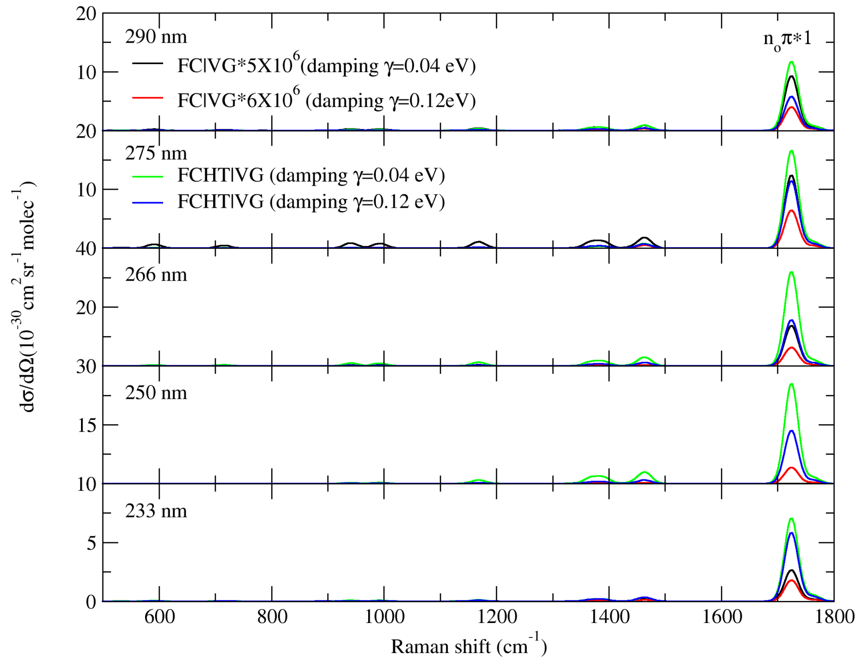

Figure S17: Vibrational resonance Raman spectra of Thymine with the predictions of the FC|VG and FCHT|VG for the first n$_O\pi^*$,obtained in CAM-B3LYP/6-311G+(d,p), computed with damping $\gamma = 0.04$ eV and 0.12 eV.

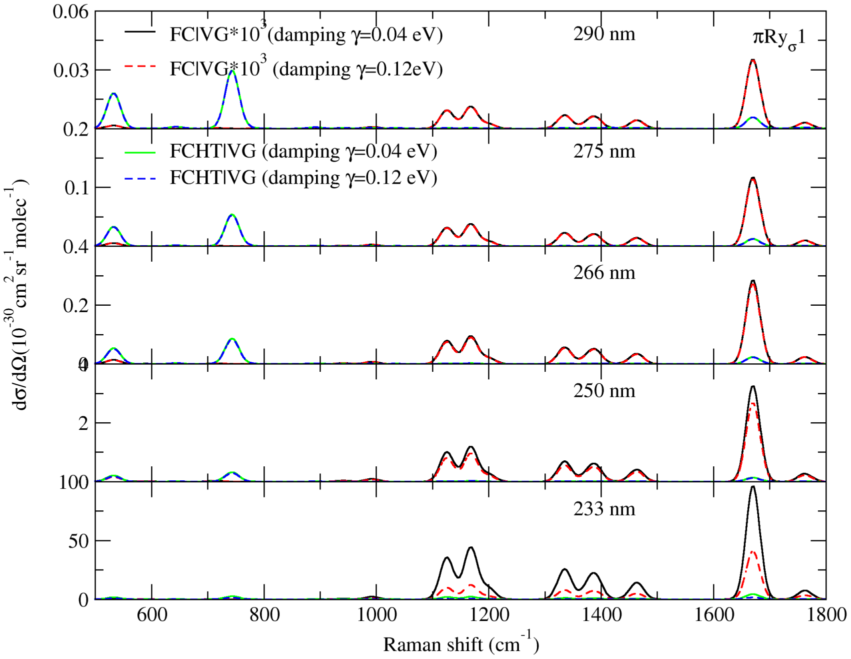

Figure S18: Vibrational Resonance Raman Spectra of Thymine with the predictions of the FC|VG and FCHT|VG for the first $\pi Ry_{\sigma}1$,obtained in CAM-B3LYP/6-311G+(d,p), computed with damping γ = 0.04 eV and 0.12 eV.

S4.8.2 Comparison of LVC and FCHT|VG predictions for the A" mode 17

Figure S19 should be compared with Figure 7 in the main text. The only difference is that whereas in the main text FCHT approximation was only applied for the quasi-dark states n$_O\pi^*$ and $\pi Ry_{\sigma 1}$, whereas the bright state $\pi\pi_1^*$ was treated at FC level (and therefore gives no contribution), here we applied the FCHT approximation also to state $\pi\pi_1^*$. The result is the appearance of a spurious and huge signal arising from $\pi\pi_1^*$, leading to a total spectrum VG Int or VG Sum that is $\sim$ 10^3-times too strong with respect to what predicted by LVC model. This finding documents the possible artefacts arising from the usage of FCHT approximation for bright states and strong coupling regimes.

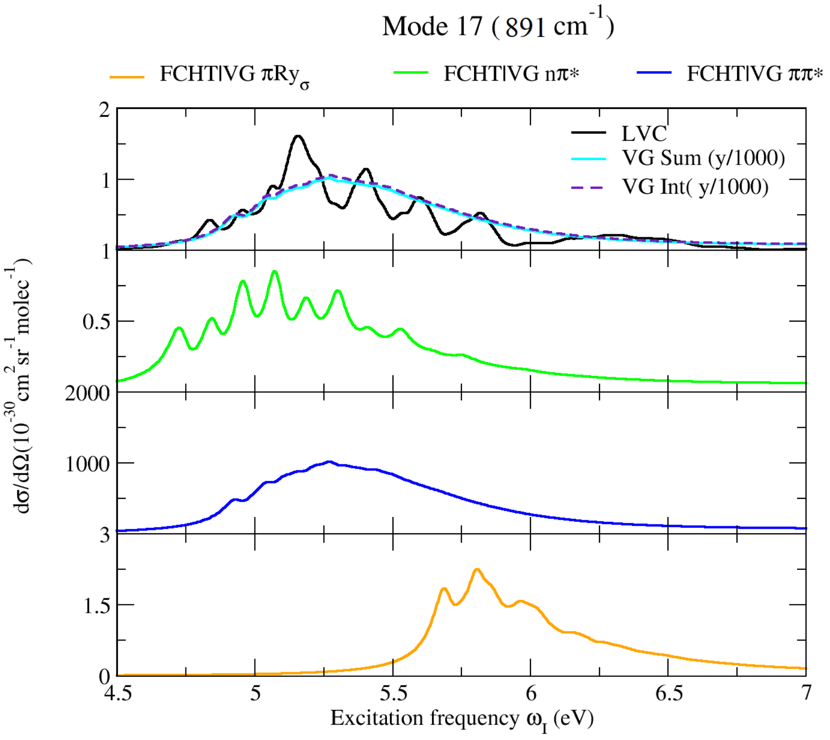

Figure S19: The Raman excitation profile of A" mode 17 of Thymine. Comparison of the predictions with LVC and with FCHT|VG computations for the first three excited states, computed with damping $\gamma = 0.04$ eV.

S5 Computations in water

Table S2: Symmetry, vertical excitation energies E_{gf} (eV), oscillator strengths (δ_{OPA}) of the first three excited states for Thymine, calculated with CAM-B3LYP/6-311G+(d,p) level in gas phase and water (considering PCM in both equilibrium and nonequilibrium regimes).

STATE	gas					
	Sym.	E_{gf}(eV)	δ_{OPA}	Trans.	Char.	Coeff.
S_1	A"	5.14	0.00	H-1→L	$n_O\pi_1^*$	0.63
S_2	A'	5.31	0.19	H→L	$\pi\pi_1^*$	0.69
S_3	A"	5.94	0.0006	H→L+1	$\pi Ry_\sigma 1$	0.69
S_4	A"	6.47	0.00	H-1→L+4	$n_O\pi_2^*$	0.40
				H-3→L+4		0.38
S_5	A'	6.67	0.055	H-2→L	$\pi\pi_2^*$	0.69
S_6	A'	6.73	0.22	H→L+4	$\pi\pi_3^*$	0.66
S_7	A"	6.78	0.0013	H→L+3	$\pi Ry_\sigma 2$	0.60
STATE	water PCM (non-equilibrium)					
	Sym.	E_{gf}(eV)	δ_{OPA}	Trans.	Char.	Coeff.
S_1	A'	5.21	0.25	H→L	$\pi\pi_1^*$	0.69
S_2	A"	5.35	0.00	H-1→L	$n_O\pi_1^*$	0.63
S_3	A"	6.32	0.0017	H→L+1	$\pi Ry_\sigma 1$	0.68
S_4	A'	6.52	0.28	H→L+4	$\pi\pi_2^*$	0.69
S_5	A"	6.60	0.00	H-3→L+4	$n_O\pi_2^*$	0.44
				H-1→L+4		0.42
S_6	A'	6.79	0.13	H-2→L	$\pi\pi_3^*$	0.68
S_7	A"	6.93	0.0003	H→L+2	$\pi Ry_\sigma 2$	0.60
STATE	water PCM (equilibrium)					
	Sym.	E_{gf}(eV)	δ_{OPA}	Trans.	Char.	Coeff.
S_1	A'	5.06	0.38	H→L	$\pi\pi_1^*$	0.70
S_2	A"	5.35	0.00	H-1→L	$n_O\pi_1^*$	0.63
S_3	A"	6.30	0.0033	H→L+1	$\pi Ry_\sigma 1$	0.68
S_4	A'	6.41	0.41	H→L+4	$\pi\pi_2^*$	0.69
S_5	A"	6.60	0.00	H-3→L+4	$n_O\pi_2^*$	0.44
				H-1→L+4		0.42
S_6	A'	6.71	0.18	H-2→L	$\pi\pi_3^*$	0.68
S_7	A"	6.91	0.0003	H→L+2	$\pi Ry_\sigma 2$	0.59

S5.1 Absorption spectrum with "single-state" approaches

S5.1.1 Contribution of $\pi\pi^*$ 1 in water

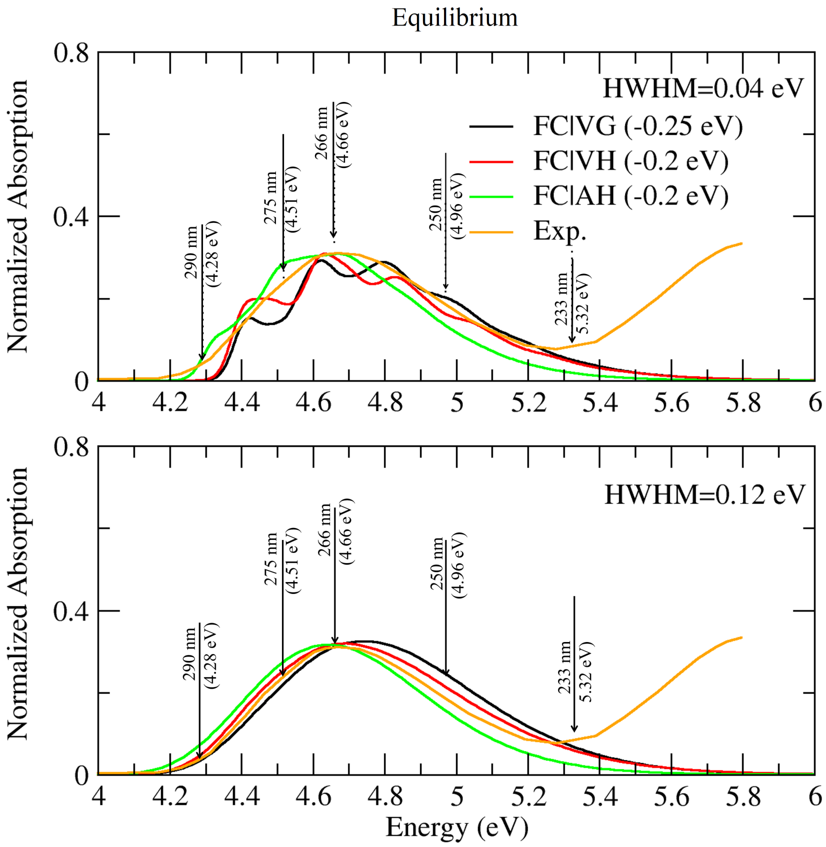

Figure S20: Absorption spectra of Thymine in water (equilibrium PCM) with the predictions of FC|VG, FC|VH, and FC|AH for the first $\pi\pi_1^*$, convoluted with a Gaussian of HWHM = 0.04 eV and HWHM = 0.12 eV. Experimental data[S5] is in aqueous solution.

It is worthy to highlight that spectra in Figure S20 were computed modelling the $\pi\pi^*1$ PES with equilibrium calculations, the most natural option when computing excited-state frequencies with Gaussian 16 (necessary for AH and VH models). For this reason the discrepancy with respect to experiment is just of 0.25 eV. By replacing vertical excitations computed in nonequilibrium regime (see Table S2), the most appropriate choice for ABS computations,[S6] the discrepancy with experiment raises up to 0.4 eV. These differences can be better appreciated inspecting the following figures where we focus on the VG model only. Specifically in Figure S21 we report

the spectrum computed summing the contribution of all the lowest-energy 7 states with PCM equilibrium computations. The same calculation was repeated in Figure S22, simply shifting the vertical excitation energy according to PCM nonequilibrium computations. It can be clearly seen that in order to overlap with the position of the first experimental band, the spectra computed in equilibrium regime must be shifted by $\sim$ 0.25 eV, whereas those in nonequilibrium regime need a larger shift by $\sim$ 0.4 eV.

S5.1.2 Adding the contribution of $\pi\pi^*$ 2 and $\pi\pi^*$3 in water

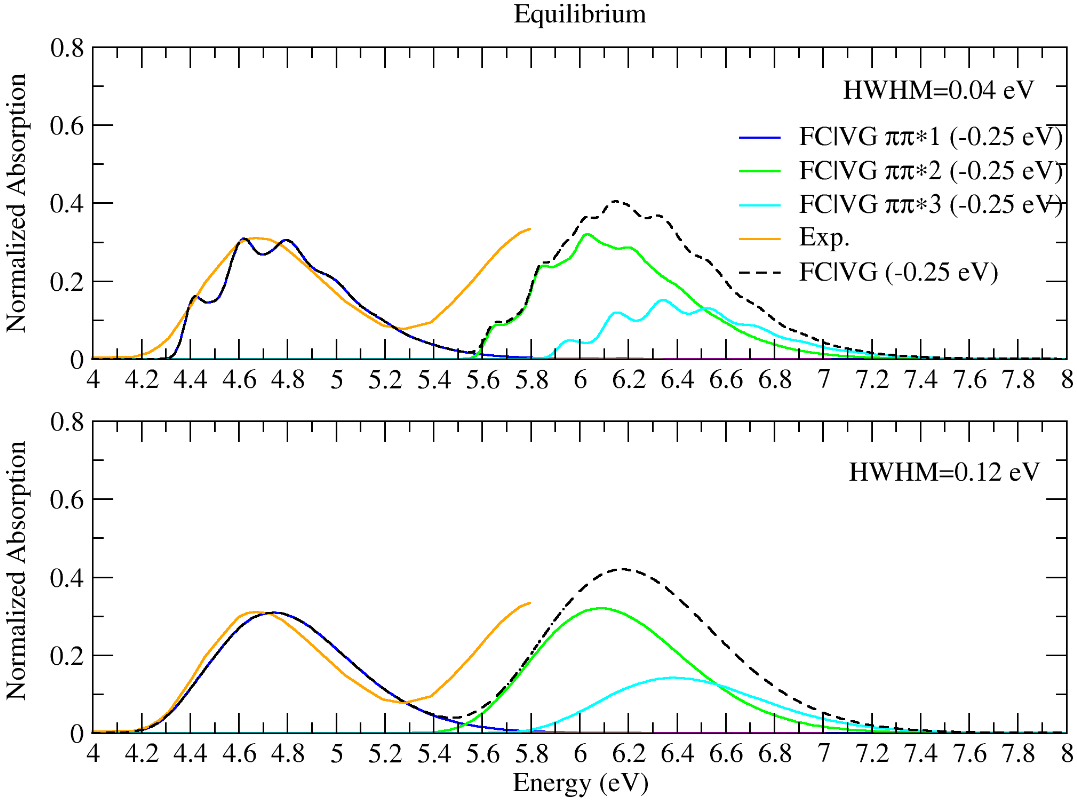

Figure S21: Absorption spectra of Thymine with the predictions of FC|VG for the first three $\pi\pi^*$ states in water (PCM equilibrium), convoluted with a Gaussian of HWHM = 0.04 eV and HWHM = 0.12 eV. Experimental data[S5] is in aqueous solution.

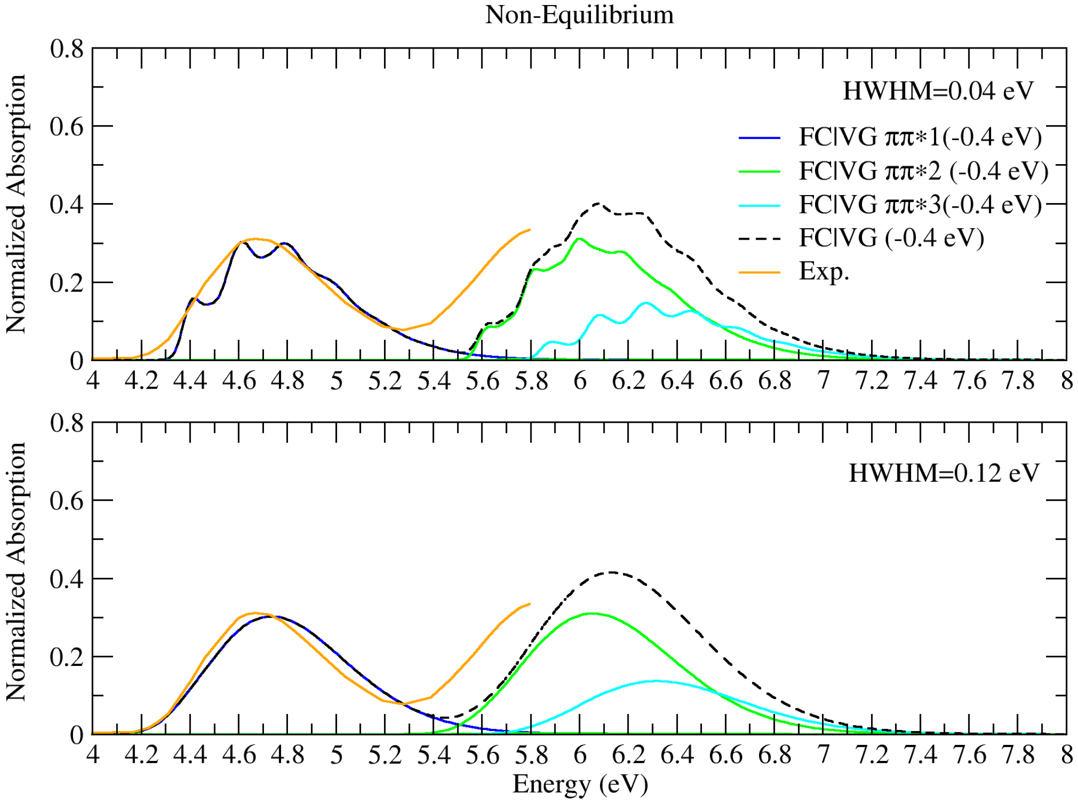

Figure S22: Absorption spectra of Thymine in water with the predictions of FC|VG for the first three $\pi\pi^*$ states (obtained from those in the previous figure by recomputing vertical transition energies with PCM nonequilibrium) , convoluted with a Gaussian of HWHM = 0.04 eV and HWHM = 0.12 eV. Experimental data[S5] is in aqueous solution.

S5.2 Vibrational resonance Raman spectra with "single-state" approaches

S5.2.1 The contribution of $\pi\pi^*$ 1 in water

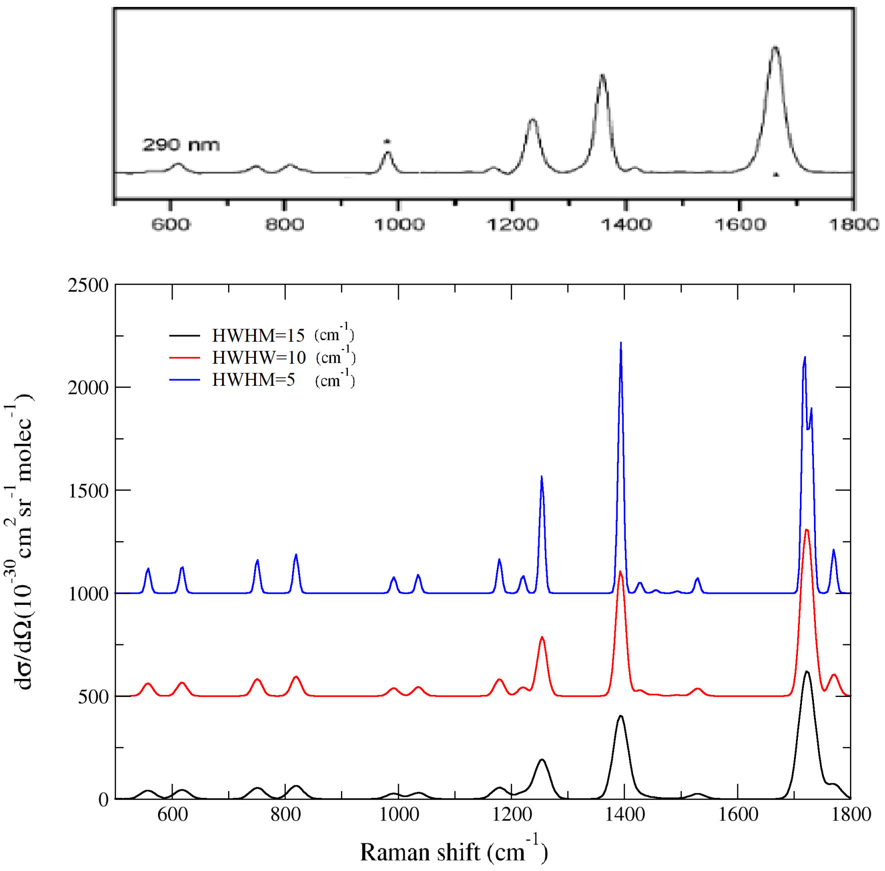

Figure S23: Vibrational resonance Raman Spectra of Thymine in water (PCM equilibrium) with different Gaussian broadenings for the Raman shift. FC|VG predictions with CAM-B3LYP/6-311G+(d,p), computed with a damping $\gamma = 0.04$ eV. The experimental data reported as insets is reprinted with permission from[S5] in aqueous solutions. Copyright 2007 American Chemical Society.

S5.2.2 Effect of different PES models: Predictions of VH and AH models for $\pi\pi_1^*$ in water

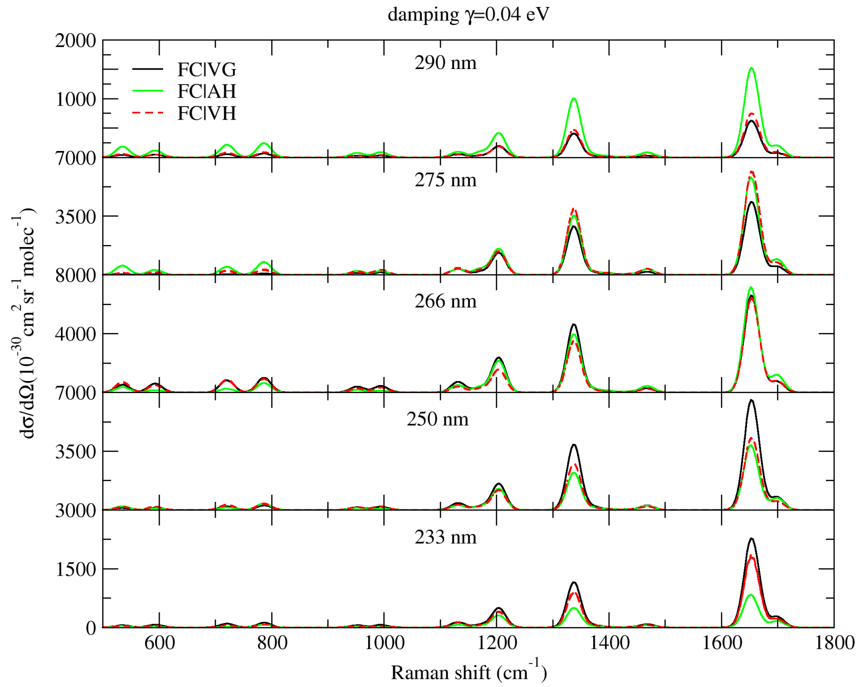

Figure S24: Vibrational resonance Raman Spectra of Thymine in water (PCM equilibrium) with the predictions of FC|VG,FC|VH and FC|AH model,obtained in CAM-B3LYP/6-311G+(d,p), computed with damping $\gamma = 0.04$ eV. The VH modes with imaginary frequencies are 1 (393.4375 i), 2 (214.9406 i), 3 (172.9273i) and 4 (117.3109i).The AH modes with imaginary frequencies are 1 (164.3319i) and 2 (120.6937i). For the treatment of these modes we followed the same strategy described in the caption of Figure S9

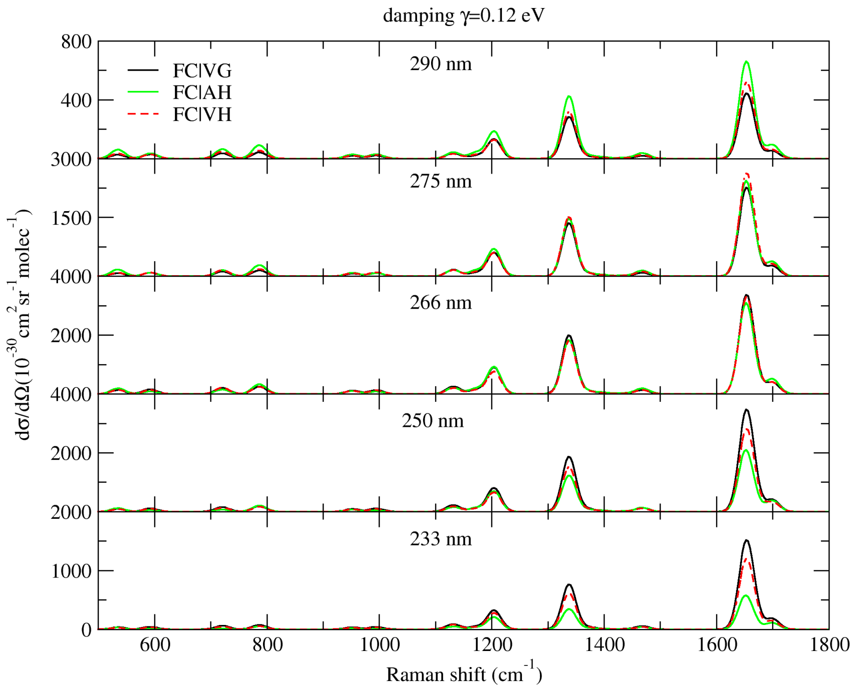

Figure S25: Vibrational resonance Raman Spectra of Thymine in water (PCM equilibrium) with the predictions of FC|VG, FC|VH and FC|AH model,obtained in CAM-B3LYP/6-311G+(d,p), computed with damping $\gamma = 0.12$ eV. The VH modes with imaginary frequencies are 1 (393.4375i), 2 (214.9406i), 3 (172.9273i) and 4 (117.3109i).The AH modes with imaginary frequencies are 1 (164.3319i) and 2 (120.6937i).

S5.2.3 Vibrational resonance Raman spectra in water adding the contribution of $\pi\pi^*2$ and $\pi\pi^*3$

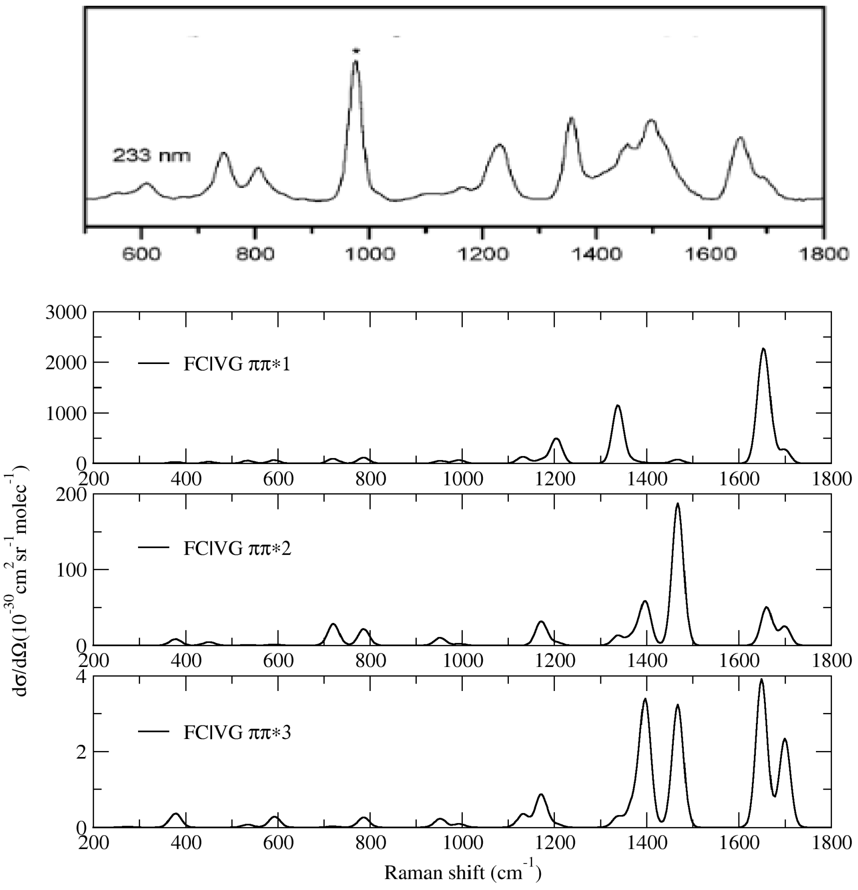

Figure S26: Vibrational resonance Raman spectra of Thymine in water (PCM equilibrium) with the predictions of FC|VG model in water, excited at 5.32 eV (233 nm), obtained with CAM-B3LYP/6-311G+(d,p), computed with damping γ = 0.04 eV. The experimental data reported as insets is reprinted with permission from[S5] in aqueous solutions. Copyright 2007 American Chemical Society.

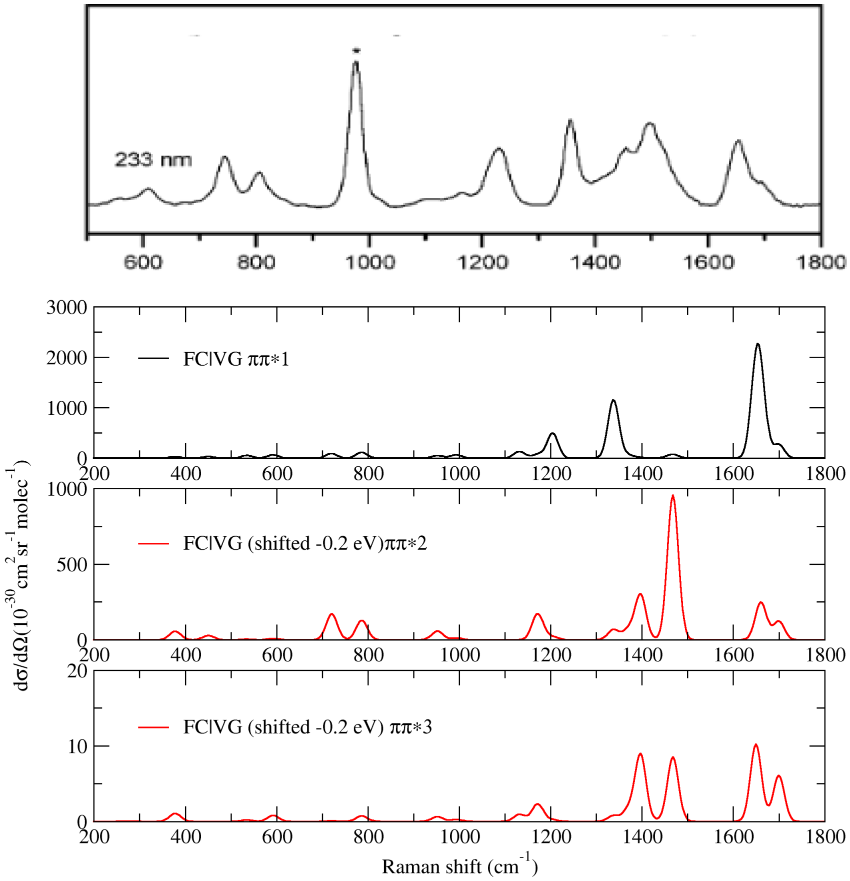

Figure S27: Vibrational resonance Raman spectra of Thymine in water (PCM equilibrium) with the predictions of FC|VG model, corresponding to an experimental excitation at 5.32 eV (233 nm), obtained with CAM-B3LYP/6-311G+(d,p), computed with damping $\gamma = 0.12$ eV. T The energy of $\pi\pi^{*}2$ and $\pi\pi^{*}3$ are artificially red-shifted by 0.2 eV. The experimental data reported as insets is reprinted with permission from[S5] in aqueous solutions. Copyright 2007 American Chemical Society.

S5.3 Tests of the impact of the PCM cavity-size parameter α on the absorption and resonance Raman spectra

In this section we investigate the impact on the results of the size of the PCM cavity. In particular we repeated the FC|VG vibronic computations considering only the $\pi\pi_1^*$ in water using three values for electrostatic scaling factor α (1.0, 1.1, and 1.2) by which the sphere radii adopted to build up the PCM cavity are multiplied. The default value employed for all the other computations reported in this work is α=1.1.

S5.3.1 Absorption spectrum of $\pi\pi^*$ 1 in water with different α

Figure S28 shows that the value of α has basically no impact on the shape of the absorption spectrum whereas it shifts sligtly its position. Notice in fact from the inset that the spectra with α=1.0, 1.1 and 1.2 have been red-shifted by a different extent in order to best overlap with the experimental spectrum. These same different shifts have been adopted to determine the excitation energies for the vRR spectra reported in the following section.

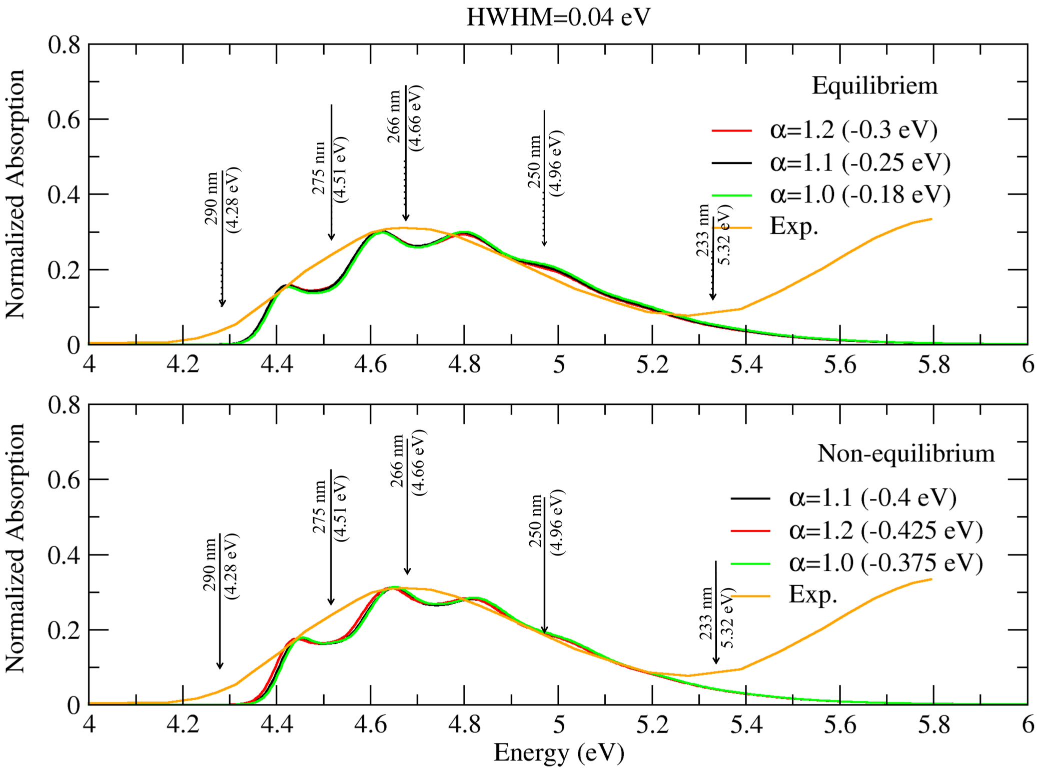

Figure S28: Absorption spectra of Thymine in water (equilibrium and non-equilibrium PCM) with the predictions of FC|VG and different PCM α values for the first $\pi\pi_1^*$, convoluted with a Gaussian of HWHM = 0.04 eV. Experimental spectrum in aqueous solution is taken from ref.[S5]

S5.3.2 Vibrational resonance Raman spectra of $\pi\pi^*$ 1 in water with different α

Figures S29 and S30 report the vRR spectra in water computed respectively with equilibrium and nonequilibrium regimes. The impact of the value of the parameter α is generally weak apart from the bands above 1600 cm^{-1}. More specifically the CO stretching is very sensitive to the solvent effects and therefore to the α value. For the smallest PCM cavity (α=1.0) this causes a red-shift of the CO stretching and therefore a splitting of the intense vRR band $>$ 1600 cm^{-1} that deteriorates the agreement with the experiment. Further discussion on this point is postponed to next subsection.

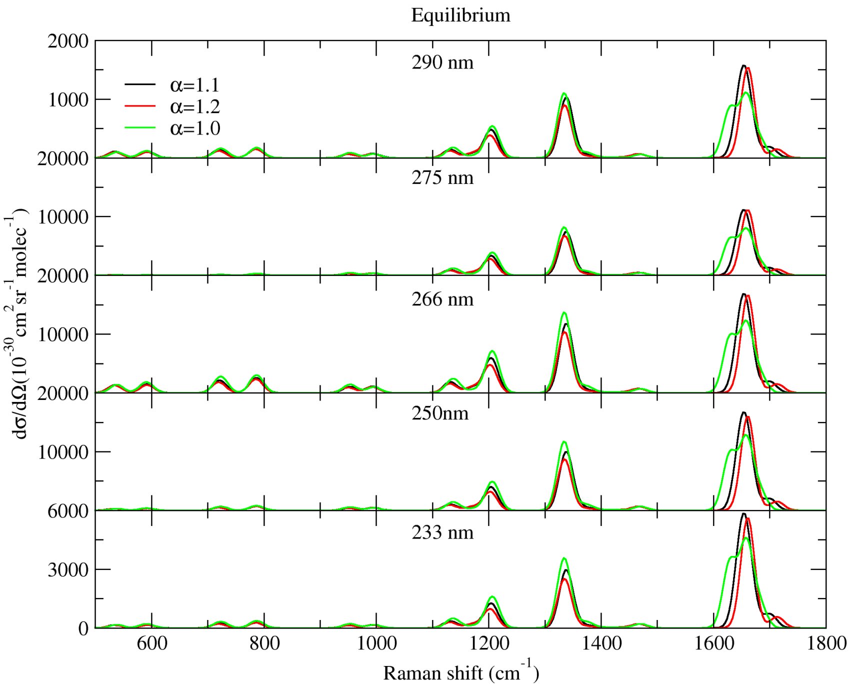

Figure S29: Vibrational resonance Raman Spectra of Thymine in water (PCM equilibrium) with different α. FC|VG predictions with CAM-B3LYP/6-311G+(d,p), computed with a damping γ = 0.04 eV.

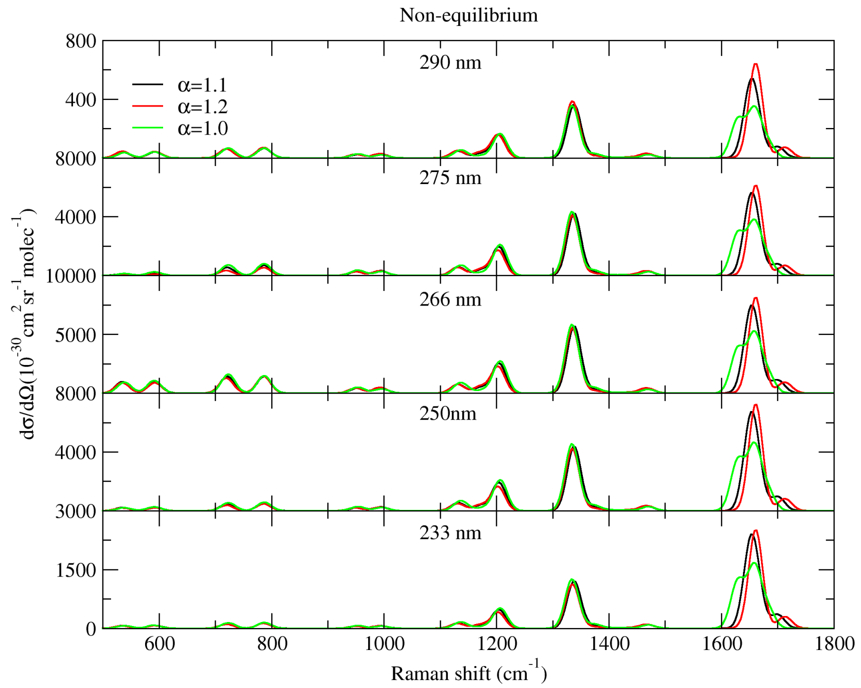

Figure S30: Vibrational resonance Raman Spectra of Thymine in water (PCM non-equilibrium) with different α. FC|VG predictions with CAM-B3LYP/6-311G+(d,p), computed with a damping $\gamma = 0.04$ eV.

S5.3.3 Raman excitation profiles of $\pi\pi^*$ 1 in water with different α

An even more stringent analysis of the effect of the PCM cavity parameter α can be obtained by investigating the Raman excitation profiles. They are reported for the fundamental of three modes in Figure S31 considering the three values of α=1.0, 1.0, 1.2. Shapes of the Raman profiles are similar but their intensities can change remarkably. This is particularly true for mode 31 and 32 for which the Raman profiles have similar intensities for α 1.0 and 1.1. On the contrary, for α=1.2 the band of mode 32 becomes extremely weak and the one of mode 31 becomes more intense. This phenomenon can be explained looking at the data in Tables S3 and S4. As we discussed above, the CO stretching is more sensitive to the α parameter and its frequency red-shifts at the decrease of α. For this reason for α=1.0 and 1.1 mode 31 is essentially the CO and mode 32 is the CC stretching, whereas for α=1.2 the two modes are very mixed (look at the Duschinsky elements in Table S4). In this case the mixed CC and CO mode 31 exhibits a large displacement between the ground state and $\pi\pi_1^*$ geometries and therefore a large Raman intensity, whereas the displacement along mode 32 is small and consequently its contribution to the Raman intensity becomes negligible (Figure S31). These two effects compensate each other so that the total impact on the vRR spectra reported in Figures S29 and S30 is small. In gas-phase the CO stretching blue-shifts further and becomes mode 32 whereas mode 31 is the CC stretching. Table S3 gives a more clear view of this phenomenon. With respect to the frequency of the CC stretching, the frequency of the CO stretching is remarkably larger in gas-phase (causing the splitting of the band > 1600 cm^{-1} discussed in Figure 2 in the main text), similar in water for α=1.1 and 1.2, and smaller for α=1.0 causing again a splitting of the vRR band > 1600 cm^{-1} as observed in the previous section.

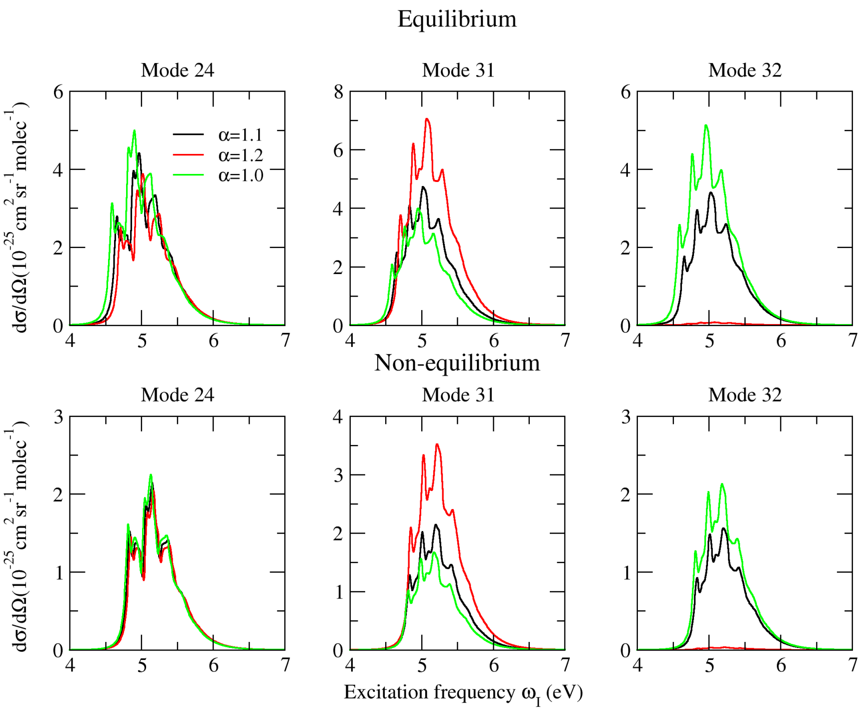

Figure S31: Raman excitation profiles of three modes of Thymine (notice the excitation frequencies have not been shifted), computed with FC|VG model and a damping $\gamma = 0.04$ eV, obtained in water with Cam-B3LYP/6-311G+(d,p) and different values of the PCM α parameter.

Table S3: The ground state frequencies (cm^{-1}) for mode 31 and mode 32 computed with CAM-B3LYP/6-311G+(d,p) in gas and in water (PCM) with different α cavity size parameter.

Frequency	Water			Gas
	α=1.0	α=1.1	α=1.2	-
31	1696.92	1718.64	1730.29	1740.28
32	1727.43	1730.75	1737.06	1796.53

Table S4: Analysis of the similarity of modes 31 and 32 computed in water, with different values for the cavity size parameter α, and in gas phase, obtained by reporting the square of the Duschinsky matrix elements with respect to the modes computed in water with α=1.1, taken as a reference

	Water						Gas	
	α=1.0		α=1.1		α=1.2		-	
	31	32	31	32	31	32	31	32
31	0.97	0.03	1.0	0	0.46	0.54	0.06	0.86
32	0.03	0.97	0	1.0	0.54	0.46	0.93	0.07

For water with α=1.0 and α=1.1, mode 31 corresponds to CO stretching and mode 32 corresponds to CC stretching.
For gas phase, mode 31 corresponds to CC stretching and mode 32 corresponds to CO stretching.

References

(S1) Avila Ferrer, F. J.; Santoro, F. Comparison of vertical and adiabatic harmonic approaches for the calculation of the vibrational structure of electronic spectra. *Phys. Chem. Chem. Phys.* **2012**, *14*, 13549–13563.

(S2) Santoro, F.; Cerezo, J. $\mathcal{FC}classes3$, a code for vibronic calculations. Available at `http://www.iccom.cnr.it/en/fcclasses`. 2022, last accessed on 30 Aug. 2022.

(S3) von Cosel, J.; Cerezo, J.; Kern-Michler, D.; Neumann, C.; van Wilderen, L. J. G. W.; Bredenbeck, J.; Santoro, F.; Burghardt, I. Vibrationally resolved electronic spectra including vibrational pre-excitation: Theory and application to VIPER spectroscopy. *J. Chem. Phys.* **2017**, *147*, 164116.

(S4) de Souza, B.; Farias, G.; Neese, F.; Izsák, R. Efficient simulation of overtones and combination bands in resonant Raman spectra. *J. Chem. Phys.* **2019**, *150*, 214102.

(S5) Yarasi, S.; Brost, P.; Loppnow, G. R. Initial excited-state structural dynamics of thymine are coincident with the expected photochemical dynamics. *J. Phys. Chem. A* **2007**, *111*, 5130–5135.

(S6) Santoro, F.; Improta, R.; Lami, A.; Bloino, J.; Barone, V. Effective method to compute Franck-Condon integrals for optical spectra of large molecules in solution. *J. Chem. Phys.* **2007**, *126*, 084509.
